# Wheat yield and grain-filling characteristics due to cultivar replacement in the Haihe Plain in China

**DOI:** 10.3389/fpls.2024.1374453

**Published:** 2024-07-08

**Authors:** Xuecheng Zhang, Xiaoli Liu, Li Wang, Qiancheng Zhao, Yang Yu, Ruirui Du, Yadong Xu, Wenchao Zhen, Yandong Wang

**Affiliations:** ^1^ College of Agronomy, Hebei Agricultural University, State Key Laboratory of North China Crop Improvement and Regulation, Key Laboratory of North China Water–saving Agriculture, Ministry of Agriculture and Rural Affairs, Key Laboratory of Crop Growth Regulation of Hebei Province, Baoding, Hebei, China; ^2^ College of Agricultural Sciences, Zhengzhou University, Zhengzhou, Henan, China

**Keywords:** Haihe Plain, winter wheat, cultivar replacement, grain yield, grain-filling characters

## Abstract

**Background:**

The Haihe Plain plays an important role in wheat production and food security in China and has experienced continuous cultivar replacement since the 1950s.This study assessed the evolution of the yield and grain-filling characteristics of the main winter wheat cultivars in the Haihe Plain over the last seven decades (1950s to date).

**Methods:**

Cultivar characterization indicated that the increase in yield was negatively affected by spike number and positively affected by the number of kernels per spike before the 2000s and kernel weight after the 2000s. Field trials were conducted across two ecological zones over two consecutive wheatgrowing seasons. The results showed that genetic gains in grain yield, spike number, and kernel weight during 1955 to 2021 were 0.629%, 0.574%, and 0.332% year–1 on a relative basis or 39.12 kg ha^–1^, 24,350 hm^–2^, and 0.15 g year^–1^ on an absolute basis, respectively. However, the increase in the kernel number per spike was not significant. Moreover, cultivar replacement explained 25.6%, 12.8%, and 37.5% of the total variance in grain yield, spike number, and kernel weight, respectively. In summary, during the initial grain-filling stage, wheat cultivar replacement led to the shortening of grain-filling duration and rapid grain-filling rate. However, a longer active grain-filling duration was produced by prolonged durations of rapid and late grain-filling. Additionally, the experimental year had a greater effect on the kernel number, which explained 53.2% of the total variance. Ultimately, modern wheat cultivars had a greater kernel weight.

**Results:**

Although the increase in kernel weight has affected grain yield during cultivar replacements in the Haihe Plain, the potential for further yield increase through kernel weight enhancement alone is limited. Consequently, future breeding efforts and cultivation practices should focus on improving spike traits and canopy architecture to enhance productivity.

## 1 Introduction

Wheat (*Triticum aestivum* L.), one of the most extensively cultivated crops worldwide, is a vital component of the global food security mosaic and accounts for one-fifth of the world’s total calories (Reynolds et al., 2011). The Haihe Plain, situated in the northern part of the Huanghuaihai Plain in China, is an important wheat-producing region. In 2021, the area dedicated to wheat cultivation was 2,247 million hectares, yielding a total production of 14,691 million tons (NBS, 2022). Since 1949, the Huanghuaihai wheat region has undergone seven to eight cultivar updates, leading to substantial advancements. In the 1960s, wheat yields were below 750 kg ha^–1^ due to stripe rust and inadequate cultivation techniques. However, the 1970s marked a significant improvement with the introduction of high-yield potential and rust-resistant cultivars, increasing yields to 1,500 kg ha^–1^. The 1980s brought further advancements, including improved lodging resistance, resulting in yields exceeding 3,750 kg ha^–1^. During the 1990s, the development of high-yielding, multi-resistant cultivars pushed yields to between 4,500 and 5,400 kg ha^–1^. In the 21st century, the focus has shifted to cultivars that offer high productivity, quality, and efficiency (Chen et al., 2008). Therefore, clarity regarding the evolutionary law of yield and agronomic traits of cultivar replacement for the breeding, cultivation, and management of winter wheat in the Haihe Plain is needed.

Since the Green Revolution, the introduction of semi-dwarf cultivars has led to dramatic increases in wheat yield (Shiferaw et al., 2013). Research has shown that reductions in plant height are key traits associated with genetic gains in wheat, and the genetic gain in wheat plant height was −0.5% (Zhang et al., 2020; Wang et al., 2023). The replacement of wheat cultivars has optimized the distribution of dry matter. Root systems, as absorbing organs, are smaller in modern cultivars (Fang et al., 2021). Modern cultivars exhibit a decrease in total root length and root biomass (BIO), with total root length per plant following a non-linear decline pattern with the year of release, decreasing from 16.2 m plant^−1^ to 10.1 m plant^−1^, and total root BIO declining linearly with the year of release at a rate of 0.058 g plant^−1^ year^−1^ (Ludlow and Muchow, 1990; Aziz et al., 2016). The smaller root systems of modern wheat cultivars enhance population performance, increase the harvest index (HI), and consequently boost yield, with the root-to-shoot ratio negatively correlated with yield (*r* = −0.77) and the HI positively correlated with yield (*r* = 0.74) (Zhu et al., 2018). Additionally, the improvement of photosynthetic performance can also be caused by cultivar replacements. Tian et al. (2011) found that leaf area, leaf area index (LAI), and the net photosynthetic rate (*P*_n_) of flag leaves significantly increased during cultivar development. Specifically, the leaf area increased by 0.41–0.53 cm^2^ annually, with LAI gradually rising from 4.87 to 6.03. The *P*_n_ of the flag leaf also increased gradually, from 19.24 to 22.22 μmol CO_2_ m^−2^ s^−1^. This enhancement in photosynthetic performance was primarily achieved by prolonging the photosynthetic duration. The photosynthetic activity duration similarly increased with cultivar development, from 266.58 to 334.74 μmol CO_2_ m^−2^ s^−1^ day. Beche et al. (2014) demonstrated that increasing the HI and BIO led to higher grain yields. The HI increased on average by 0.24% year^−1^, while BIO also increased, with an average increase of 47.59 kg ha^−1^ year^−1^. However, as a crucial aspect of yield formation and BIO allocation, studies on the effects of variety replacement on grain-filling characteristics are still relatively rare.

The replacement of crop cultivars has a significant influence on yield. For example, Cao (2001) showed that wheat cultivars replaced once can generally increase the yield by approximately 10% The annual genetic gain in wheat yield in the northern wheat region of China was 0.48%–1.23% (Zhou et al., 2007a; Qin et al., 2019). Aisawi et al. (2015) showed that wheat kernel weight gradually increased with cultivar replacements, but kernel number per spike did not change significantly. Thapa et al. (2019) showed that, compared with spike number and kernel number per spike, kernel weight had the greatest response to water stress. Owing to the continuous increase in grain production in the Haihe Plain over the past 50 years, groundwater extraction in agricultural areas has continuously increased (Feng et al., 2013). Therefore, water conservation and drought resistance are highly desirable traits that require an increase in the kernel weight of wheat to stabilize the spike number. Wheat kernel weight mainly depends on the transport and distribution of assimilates to grains during grain formation (Wan et al., 2022). Sink strength, which includes sink size and activity, affects the ability of wheat grains to accept assimilates (Dreccer et al., 1997; Borrás et al., 2004; Hidaka et al., 2019). Meanwhile, sink activity is influenced by grain-filling characteristics, which change the grain-filling rate, thereby controlling kernel weight; therefore, sink activity is enhanced in newer wheat cultivars by changing the grain-filling characteristics to increase kernel weight (Ho, 1996). Old wheat cultivars are affected by early senescence and relatively less dry matter accumulation during the grain-filling period, resulting in lower wheat kernel weight (Acreche and Slafer, 2009).

The grain-filling characteristics have a significant impact on the yield and kernel weight. Currently, there is considerable research on wheat-filling characteristics and yield in the Haihe Plain, but most of these studies have only focused on the factors affecting grain-filling in single cultivars or cultivars in the same period (Zheng et al., 2022). However, few studies have been conducted on the evolution of the yield and grain-filling characteristics of wheat during cultivar replacement. In this study, we analyzed wheat yield progress and grain-filling characteristics of wheat cultivar replacement in the Haihe Plain over the last seven decades to (1) clarify patterns in yield and yield component changes over the last 70 years of the main winter wheat cultivars in different years in the Haihe Plain, and (2) understand the response of grain-filling characteristics to cultivar replacement.

## 2 Materials and methods

### 2.1 Study site

**Figure 1:**
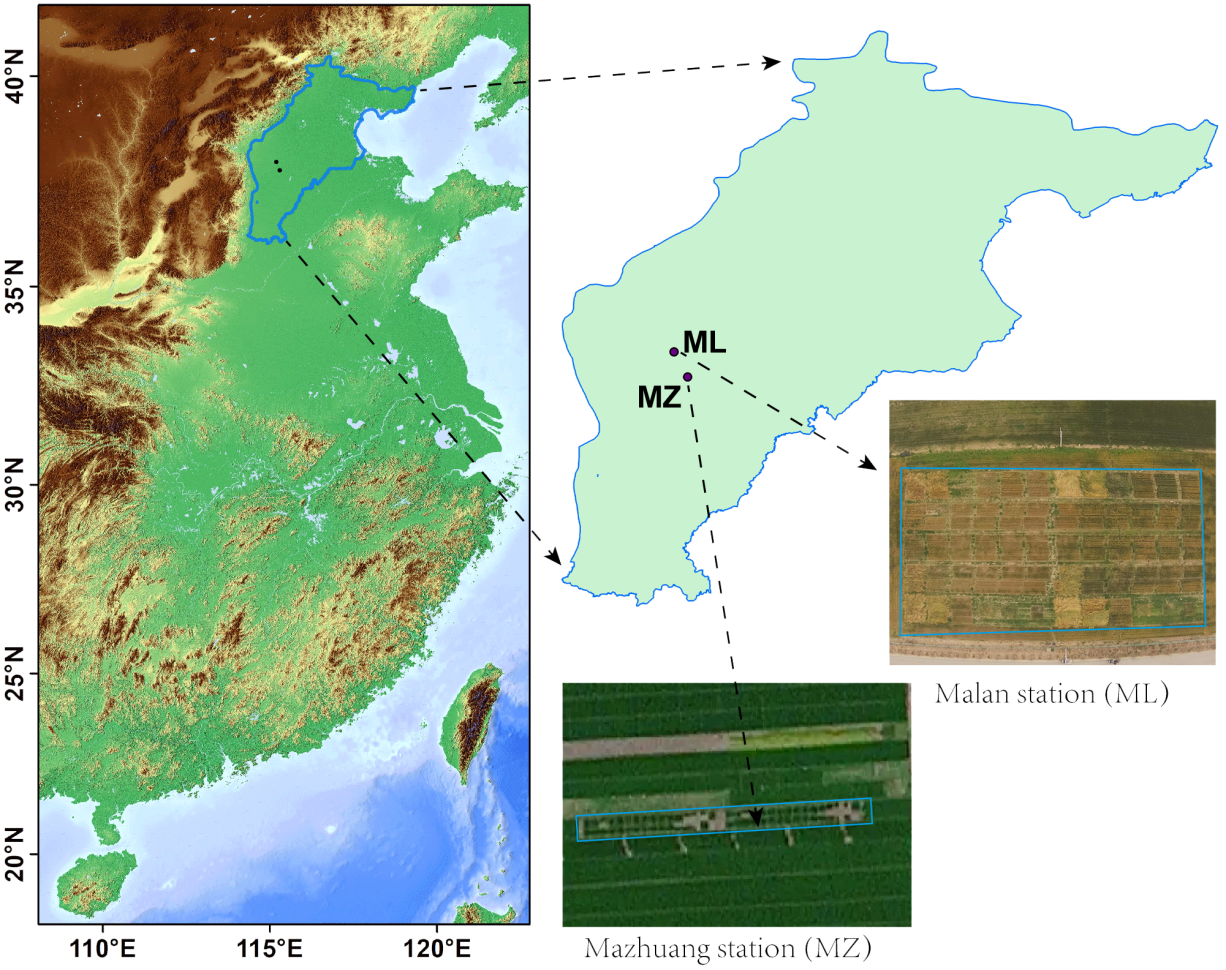
Location of experimental field sites.

This study was conducted in Hebei Province at the Mazhuang Experimental Station (MZ, 37°47′52″N, 115°18′28″E) during the 2021/2022 wheat growing season and at the Malan Experimental Station (ML, 37°58′28″N, 115°12′2″E) during the 2021/2022 and 2022/2023 wheat growing seasons (**Figure 1**). The MZ and ML experimental stations were located in the Piedmont Plain of Mt. Taihang and the Heilonggang Plain, respectively, at elevations of 53 and 37 m in the Haihe Plain (Hou and Hao, 2010). Both the Heilonggang Plain and the Piedmont Plain of Mt. Taihang have a warm temperate semi-humid continental monsoon climate, whereas the Piedmont Plain of Mt. Taihang has relatively good soil conditions (Asadi Zarch et al., 2015). The annual precipitation in 2021/2022 and 2022/2023 was 804.7 mm and 731.0 mm, respectively, with the wheat growing season receiving 174.1 mm and 181.9 mm of precipitation, respectively. The annual average temperatures for the 2021/2022 and 2022/2023 seasons were 17.2°C and 15.14°C, respectively, with the wheat growing season averaging 8.7°C and 9.4°C, respectively. During the 2022/2023 season, the lowest temperature reached −9.3°C (**Supplementary Figure S1**). The organic matter, total nitrogen, alkaline-dissolved nitrogen, available phosphorus, and potassium in the study area during the experiments were 22.4 g kg^–1^, 1.26 g kg^–1^, 131.2 mg kg^–1^, 25.8 mg kg^–1^, and 122.2 mg kg^–1^, respectively, in MZ, and 19.7 g kg^–1^, 1.09 g kg^–1^, 125.0 mg kg^–1^, 15.32 mg kg^–1^, and 101.3 mg kg^–1^, respectively, in ML.

### 2.2 Experimental design

The field arrangement followed a single-factor randomized block design with three replicates. A total of 29 winter wheat cultivars, the main cultivars in the Haihe Plain in certain years, were selected as treatments. The experimental materials were provided by the Hebei Provincial Agricultural Biological Resources Preservation Center (**Table 1**). The plot area was 30 m^2^ (6 m × 5 m; row spacing: 15 cm). Basal fertilizer consisting of 120 kg ha^–1^ N, 120 kg ha^–1^ P_2_O_5_, and 120 kg ha^–1^ K_2_O was applied in ML and MZ at Zadoks Stage 0, respectively. Wheat seeds were sown in ML and MZ at Zadoks Stage 0 (Zadoks et al., 1974) with a seeding rate of 375 seed m^−2^ and harvested in ML and MZ on 6 June and 10 June 2021 and in ML on 8 June 2022. Using the local irrigation system, irrigation was applied at the jointing at Zadoks Stage 31 and anthesis at Zadoks Stage 65 (Zadoks et al., 1974) with 90 mm of groundwater each time (Fang et al., 2017). Additionally, 120 kg ha^–1^ of N was applied during the jointing stage with irrigation, and weed and pest control were performed regularly during the growth period.

**Table 1:** Winter wheat cultivars used in this study.

Year	Cultivars	Origin	Founder parent
1955	Shijiazhuang407	Shenglimai/Yanda1817	Shenglimai/Yanda1817
1962	Beijing8	Bima4/Zaoyangmai	Bimamai/Biyumai
1965	Shijiazhuang54	Bima4/Zaoyangmai	Bimamai/Biyumai
1965	Jinan2	Bima4/Zaoyangmai	Bimamai/Biyumai
1976	Jimai1	Beijing8/H.H/Orofen	Bimamai/Biyumai
1976	Jimai2	Beijing8/H.H/Orofen	Bimamai/Biyumai
1978	Jimai3	H.HPZ41/Shijiazhuang54	Zaoyangmai
1984	Taishan1	Bima4/Zaoshu1/Orofen	Bimamai/Biyumai
1988	Jimai26	Aiganzao/Lovelin10/Jinfeng1	Lovelin10
1992	Jimai30	78–3147/Shi4414	Ourou/Zaoyangmai
1994	Jimai36	715017/Shanqian/75–78/94354	Bimamai/Biyumai
1996	Jimai38	Zhi4001/Shi4212–10	Lovelin10
1997	Heng4041	Jimai26/211–4/Jimai 26	Lovelin10
1997	Shi4185	Zhi8094/Baofeng7228/Shi84–7120	Lovelin10
1998	Han4589	Han86–4032/85Zhong47	Han4032
1998	Cang6001	Linfen6145/Jimai32	Shenglimai/Yanda1817
2001	Heng95Guan26	Heng84Guan749/Heng87–263	Lovelin10
2001	Han6172	Han4032/Zhongyin1	Han4032
2003	Shijiazhuang8	Shi91–5096/Shi9306	Lovelin10
2004	Shiluan02–1	9411/9430	Linzhangmai
2004	Hengguan35	Heng84Guan749/Heng87–4263	Lovelin10
2006	Jimai22	935024/935106	Fengchan3
2007	Shimai15	Jimai38/92R137	Lovelin10
2008	Heng4399	Han6172/Hengsui28	Han4032
2013	Shimai22	Lin8014/Jimai 38/Shi4185	Lovelin10
2019	Shinong086	Lumai14/Han6172	Fengchan3
2021	Malan1	Jimai22/Jinhe9123	Fengchan3
2021	Malan6	Gaoyou2018/ShiU09–4366	Linzhangmai

Information on winter wheat cultivars of all decades was obtained from Chen et al. (2008) and Li (2014). The information on wheat cultivars after the 2000s was from the Agricultural Dominant Cultivars and Recommended Technologies released by the Department of Agriculture and Rural Affairs of Hebei Province.

### 2.3 Sampling and laboratory analysis

#### 2.3.1 Grain-filling rate determination

At anthesis (Zadoks Stage 65), 100 spikes with consistent anthesis and similar growth without pests or diseases were marked in each plot, and samples were taken every 3 days from 7 days after anthesis according to each cultivar’s anthesis date until harvest. All grains were dried at 80°C for 72 h to a constant weight to obtain the dry weight.

**Figure 2:**
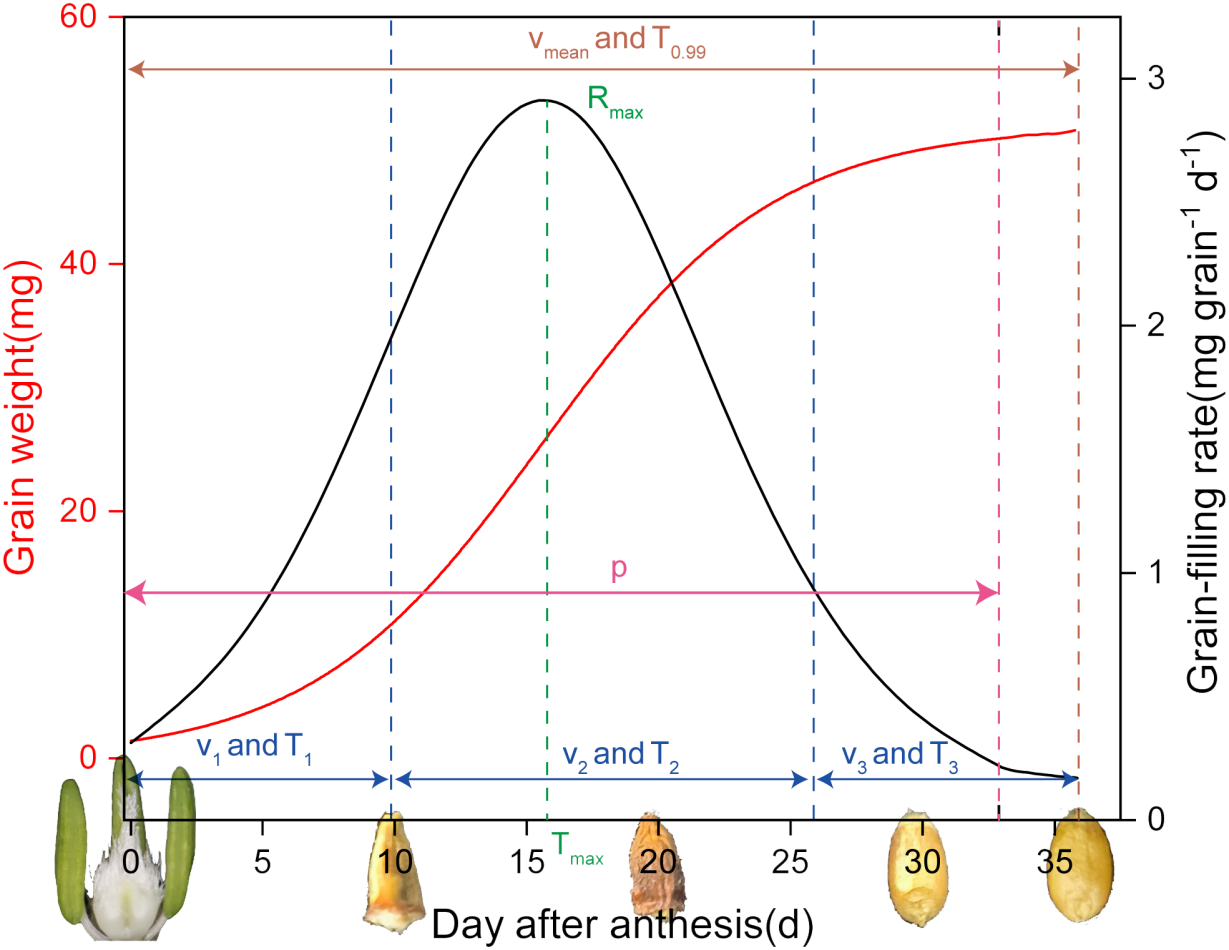
The schematic diagram of grain dry matter accumulation (red line) and grain-filling rate (black line) of winter wheat.


A logistic growth curve was fitted to the grain-filling rate using Equation 1 (**Figure 2**) (Liu et al., 2022):

(1) $$W=\frac{A}{1+Be^{-Kt}}$$

where *W* is the single kernel weight in mg, *A* is the final kernel weight (Zadoks Stage 92) in mg, *t* (d) is the number of days after anthesis, and *B* and *K* are the regression coefficients.

The maximum grain-filling rate (*R_max_*, mg grain^–1^·d^–1^) was calculated using Equation 2:


(2) $$R_{max}=\frac{KW_{max}-KW_{max}^{2}}{A}$$

The average grain-filling rate (*V_mean_*, mg grain^–1^ d^–1^) was calculated using Equation 3:


(3) $$V_{mean}=\frac{W_{3}}{T_{0.99}}$$

The effective duration of grain-filling (*T*_0.99_, d) was calculated using Equation 4:


(4) $$T_{0.99}=\frac{\left(lnB+4.59512\right)}{K}$$

The time required to reach the maximum grain-filling rate (*T_max_*, d) was calculated using Equation 5:


(5) $$T_{max}=\frac{lnB}{K}$$

The active grain-filling period (*p*, d) was calculated using Equation 6:


(6) $$p=\frac{6}{K}$$

The duration of the initial grain-filling rate (*T*_1_, d) was calculated using Equation 7:


(7) $$T_{1}=\frac{lnB-1.317}{K}$$

The average grain-filling rate at the initial grain-filling stage (*v*_1_, mg grain^−1^ d ^−1^) was calculated using Equations 8, 9, as follows:


(8) $$W_{1}=\frac{A}{1+Be^{-KT_{1}}}$$

(9) $$v_{1}=\frac{W_{1}}{T_{1}}$$

The duration of the rapid grain-filling rate (*T*_2_, d) was calculated using Equation 10:


(10) $$T_{2}=\frac{lnB+1.317}{K}-\frac{lnB-1.317}{K}$$

The average grain-filling rate of the rapid grain-filling stage (*v*_2_, mg grain^−1^ d^−1^) was calculated using Equations 11, 12, as follows:


(11) $$W_{2}=\frac{A}{1+Be^{-lnB-1.317}}$$

(12) $$v_{2}=\frac{W_{2}-W_{1}}{T_{2}}$$

The duration of the late grain-filling rate (*T*_3_, d) was calculated using Equation 13:


(13) $$T_{3}=\frac{lnB+4.59512}{K}-\frac{lnB+1.317}{K}$$

The average grain-filling rate at the late grain-filling stage (*v*_3_, mg grain^–1^ d^–1^) was calculated using Equations 14, 15, as follows:


(14) $$W_{3}=\frac{A}{1+Be^{-lnB-4.59512}}$$

(15) $$v_{3}=\frac{W_{3}-W_{2}}{T_{3}}$$

#### 2.3.2 Yield and yield components

At maturity (Zadoks Stage 92), a 1-m^2^ area from each plot was harvested to measure the spike number, thousand-kernel weight, and grain yield. The kernel number per spike (kernel number) was determined by dividing the grain yield by the product of the spike number and average kernel weight. The grain moisture content was adjusted to 13%. Data on the yield and yield components of the officially approved cultivars were obtained from the First Seed Production Industry (http://www.a-seed.cn/).

### 2.4 Statistical analysis

The logarithmic mean Divisia index (LMDI) method, which is a well-known decomposition model, was used to decompose the contribution of grain yield variation in different years to spike number, kernel number per spike, and grain weight (Xie et al., 2019). The grain yield (*ΔY*) changes from years *t−*1 to years *t* was calculated using Equation 16, as follows:


(16) $$\Delta Y=Y_{t}-Y_{t-1}=\Delta S\times \Delta G\times \Delta K$$

where, *ΔS*, *ΔG*, and *ΔK*, respectively, represent the influence of the spike number, kernel number per spike, and kernel weight on the changes of grain yield, whose relevant decomposition factors between *t* and *t−*1 was calculated using Equations 17–20, as follows:


(17) $$\Delta S=L\left(Y^{t},\;Y^{t-1})\times \ln(\frac{S_{t}}{S_{t-1}}\right)$$

(18) $$\Delta G=L\left(Y^{t},\;Y^{t-1})\times \ln(\frac{G_{t}}{G_{t-1}}\right)$$

(19) $$\Delta K=L\left(Y^{t},\;Y^{t-1})\times \ln(\frac{K_{t}}{K_{t-1}}\right)$$

where


(20) $$L(Y^{t},\;Y^{t-1})=\frac{Y_{t}-Y_{t-1}}{lnY_{t}-lnY_{t-1}}$$

The genetic gain (GN) in grain yield and its components based on cultivar release date was calculated using Equations 21, 22 (OrtizMonasterio et al., 1997):


(21) $$y_{i}=a+bx_{i}+\mu$$

(22) $$ln\left(y_{i}\right)=a+bx_{i}+\mu$$

where *y_i_* is the grain yield and its components of cultivar *i*, ln(*y_i_*) is the natural log of *y_i_*, *x_i_* is the year in which cultivar *i* was released, *a* is the estimated intercept of each equation, and *μ* is the residual error. The linear function (Equation 21) provides an estimate of the grain yield and its components increase based on cultivar release date in absolute terms (i.e., *b* measures grain yield and its components gain), while the logarithmic function (Equation 22) gives the relative grain yield and its components increase (d*y_i_*/d*x_i_*) such that 100*b* estimates the percentage grain yield and its components gain per year. All parameters were estimated using a restricted maximum likelihood linear mixed effects model (RMLM). The RMLM was also used to evaluate the effects of cultivar replacement and planting location on the grain yield, yield components, and grain-filling parameters of winter wheat. Winter wheat cultivar nested in the founder parent as a random effect for a lack of independence within cultivar and founder parent between year of cultivar release, experimental year, and site. The RMLM was conducted in the “*lme4*” package of R (version 4.2.1) (Bates et al., 2015). The variances of each fixed variable, random variable, and unexplained factor were calculated using variance decomposition, which was performed in the “*partR2*” package of R (Stoffel et al., 2020). Additionally, redundancy analysis (RDA) was conducted using the “*vegan*” package of R (Oksanen et al., 2023), with the grain-filling parameters as independent variables, and cultivar replacement and experimental location as dependent variables. All figures were generated using ArcGIS 10.2, OriginPro 2021 and the “*ggplot2*” package in R (Wickham, 2016).

## 3 Results

### 3.1 Effect of yield components on yield during cultivar replacement

**Figure 3:**
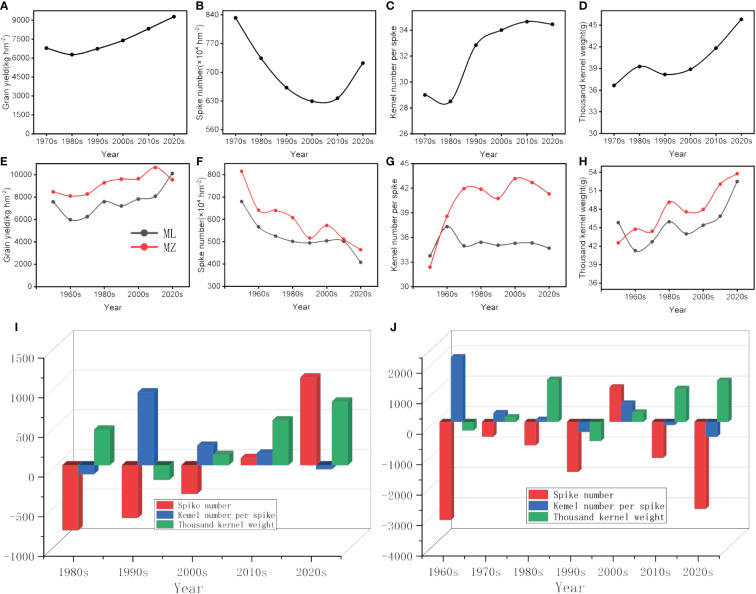
Wheat yield and its components in different years, and LMDI decomposition of the contribution of yield components to yield. **(A–D, I)** Wheat yield, its components, and LMDI decomposition of official cultivar approval data; **(E–H, J)** wheat yield, its components, and LMDI decomposition of field data.

The results of the official cultivar approval showed that the yield of wheat increased, the spike number per unit area decreased, and the kernel number per spike and kernel weight increased over the analyzed timespan (**Figures 3A–D**). The LMDI results indicated that the spike number had a negative contribution yield increase, except in the 2020s. Kernel number had a greater positive contribution to yield before the 2000s, while the kernel weight contributed positively to yield after the 2000s. Notably, the contribution of kernel weight in the 2020s increased by 43.95% compared to the 1980s (**Figure 3I**). Similarly, field experiment results showed that the yield of main winter wheat cultivars in the Haihe Plain has increased from the 1950s to the present. This increase in yield was accompanied by a decrease in spike number, and an increase in kernel number per spike and kernel weight (**Figures 3E–H**). According to the LMDI results, except for the 2000s, the spike number consistently had a negative contribution to yield increase. The LMDI indexes for the spike number were −3,222.1, −486.7, −767.7, −1,642.5, 1,137.1, −1,183.44, and −2,858.01 from the 1960s to the 2020s, respectively. Kernel number had a greater positive contribution before the 2000s, and the LMDI indexes for the kernel number were 2,126.3, 289.9, 72.7, −333.7, 605.3, −105.7, and −491.9 from the 1960s to the 2020s, respectively. After the 2000s, kernel weight contributed positively to yield, and the LMDI indexes for the kernel weight were −282.8, 152.5, 1,384.4, −627.5, 310.2, 1,087.3, and 1,350.9 from the 1960s to the 2020s, respectively (**Figure 3J**).

### 3.2 Grain yield and its components

**Figure 4:**
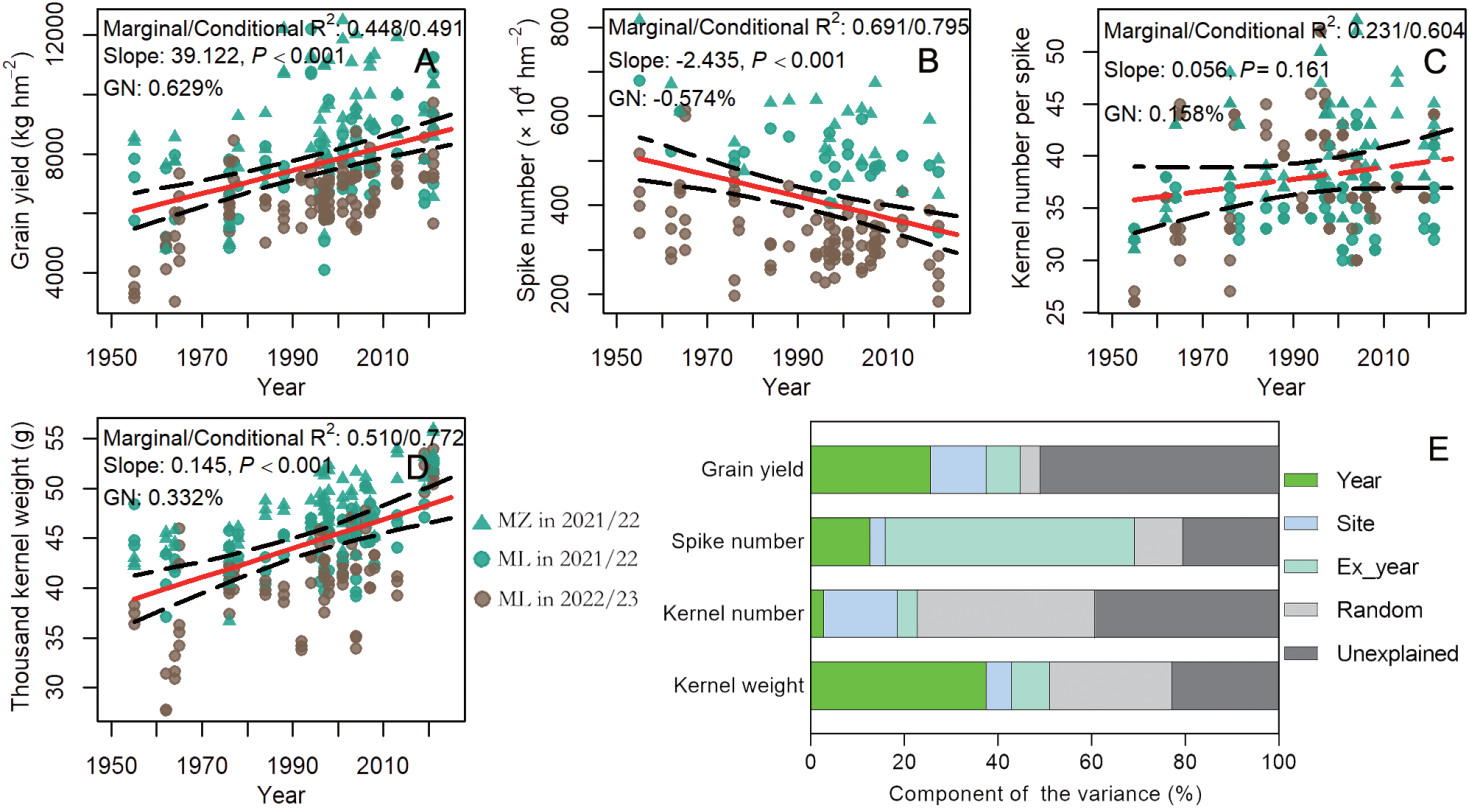
The relationship between grain yield of winter wheat and yield component with year of release by RMLM. **(A–D)** The relationship between grain yield of winter wheat and yield component with year of release; **(E)** Year, site, ex-year, random, and unexplained contributions to the variation in wheat yield and its components. The black dashed lines with edges represent the 95% confidence interval, and the red solid line is the fitted line.

In this study, the average grain yields from the 1950s to the 2020s were 6,311.1, 6,029.8, 6,299.1, 7,742.8, 7,662.2, 8,146.3, 8,536.5, and 9,014.2 kg ha^–1^, respectively. The average spike number was 638.7, 501.7, 527.4, 509.5, 432.9, 461.1, 453.1, and 373.7 ×10^4^ ha^–1^, respectively, from the 1950s to the 2020s. The average kernel number per spike was 32.8, 36.4, 35.9, 38.3, 39.9, 37.9, 39.1, and 39.4, respectively, from the 1950s to the 2020s. The average thousand kernel weight was 41.9, 39.6, 42.4, 43.5, 44.8, 44.9, 48.4, and 52.7 g, respectively, from the 1950s to the 2020s. The results of RMLM showed that grain yield significantly increased by 39.12 kg ha^–1^ and kernel weight significantly increased by 0.15 g year^–1^, respectively, spike number significantly declined by 24,350 hm^–2^ year^–1^ (*p* < 0.001), but the kernel number per spike did not significantly increase (*p* > 0.05) (**Figures 4A–D**). Genetic gain refers to the improvement in average genetic value in a population or the improvement in average phenotypic value due to selection within a population over cycles of breeding. In this study, genetic gain specifically refers to the annual percentage increase or decrease in yield and yield components. GN of grain yield, spikes per unit area, kernel number per spike, and kernel weight were 0.629%, –0.574%, 0.158%, and 0.332%, respectively (**Figures 4A–D**). Furthermore, the results of variance decomposition showed that the year of release accounted for 25.6% of the variation in grain yield, whereas planted location, unexplained factors, and experimental year accounted for 11.8%, 50.9%, and 7.38% of the variation, respectively (**Figure 4E**). This finding indicates that in addition to varietal replacement, cultivation conditions, experimental year, and other factors have a large impact on grain yield. Kernel weight was affected more by the year of release, accounting for 37.5% of the variation, and spike number was affected more by the experimental year, accounting for 53.2% of the variation. Kernel number was mainly affected by the experimental location, accounting for 15.7% of the variation (**Supplementary Table S1**; **Figure 4**).

### 3.3 Grain-filling characteristics

The logistic regression model accurately captured the trends in the wheat grain-filling process (*R* ^2^ > 0.9) (**Supplementary Table S2**). Kernel weight growth showed an “S”-shaped increase in kernel weight during the grain-filling process when visualized. With the replacement of cultivars, the kernel weight of modern cultivars was higher than that of earlier cultivars (**Supplementary Figure S2A**). The grain-filling rate increased first and then decreased. The grain-filling rate indicated that the grain weight initially increased until it reached *T_max_*, the time to reach the maximum grain-filling rate, after which it began to decrease. The *T_max_* advanced with the replacement of cultivars, which were 20.3, 19.1, 20.1, 19.7, 18.9, 18.9, 18.3 and 17.1 days for the 1950s–2020s, respectively. *R_max_* also increased gradually, except in the 1950s, with values of 2.7, 2.2, 2.2, 2.1, 2.2, 2.2, 2.3, and 2.4 mg grain^–1^ d^–1^ in the 1950s–2020s, respectively (**Supplementary Figure S2B**; **Supplementary Table S3**).

**Figure 5:**
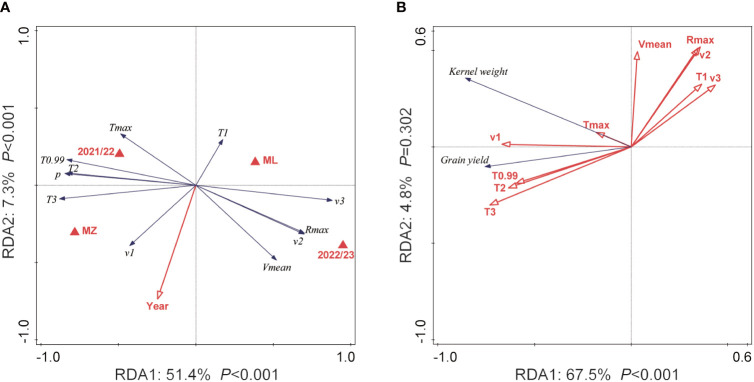
Ordination plots of the results from the redundancy analysis (RDA) to identify the relationships between winter wheat grain-filling characteristics with planting location and year of release **(A)** and between grain yield and kernel weight with grain-filling characteristics **(B)**. *R_max_* is the maximum grain-filling rate, *V_mean_* is the average grain-filling rate, *T*_0.99_ is the effective duration of the grain-filling, *T_max_* is the time to reach the maximum grain-filling rate, *p* is the active grain-filling period, *T*_1_ is the duration of the initial grain-filling rate, *T*_2_ is the duration of the rapid grain-filling rate, *T*_3_ is the average grain-filling rate in the late grain-filling stage, *v*_1_ is the average grain-filling rate at the initial grain-filling stage, *v*_2_ is the average grain-filling rate of the rapid grain-filling stage, and *v*_3_ is the average grain-filling rate at the late grain-filling stage. The red arrows represent the explanatory variables, while the black arrows represent the grain-filling parameters. The length of the arrow indicates the magnitude of influence; the smaller the angle between the arrows, the stronger the positive correlation between them. Conversely, a larger angle represents a negative correlation. The angle between the arrows and the ordination axes indicates the correlation with the axes; a smaller angle indicates a stronger correlation, while a larger angle indicates a weaker correlation.

RDA was used to determine the response of the grain-filling characteristics to the replacement of cultivars, planting locations, and experimental year. These three constraining variables explained 61.0% of the variation in grain-filling characteristics (**Supplementary Table S4**). The results showed that RDA1 accounted for 51.4% and RDA2 for 7.3% of the total variation in the grain-filling characteristics (**Figure 5A**). From left to right (MZ to ML, 2021/2022 to 2022/2023), *v*_1_ *, T*_3_ *, p, T*_2_ *, T*_0.99_, and *T_max_* decreased as the cultivation ecological environment deteriorated, whereas *v_mean_, v*_2_ *, R_max_, v*_3_, and *T*_1_ increased (**Figure 5A**). Moreover, *T*_0.99_, *T*_3_, *p*, *v_mean_*, *T*_2_, *v*_1_, *v*_2_, and *R_max_* values were positively correlated with the year of cultivar release, whereas *T_max_*, *T*_1_, and *v*_3_ were negatively correlated with the year the cultivars were released (**Figure 5A**). RDA was also used to determine the response of grain yield and kernel weight to grain-filling characteristics (**Figure 5B**). Grain yield and kernel weight explained 72.3% of the variation in grain-filling characteristics (**Supplementary Table S4**). The results showed that RDA1 accounted for 67.5% and RDA2 accounted for 4.8% of the total variation in grain yield and kernel weight, respectively, but RDA2 was not significant (*p* > 0.05). From right to left, *T*_0.99_ *, T*_2_, *T*_3_, *T_max_*, and *v*_1_ increased as grain yield and kernel weight increased, whereas *T*_1_ *, v*_2_ *, v*_3_ *, R_max_*, and *v_mean_* decreased.

### 3.4 Relationships between grain-filling characteristics and year of release

**Figure 6:**
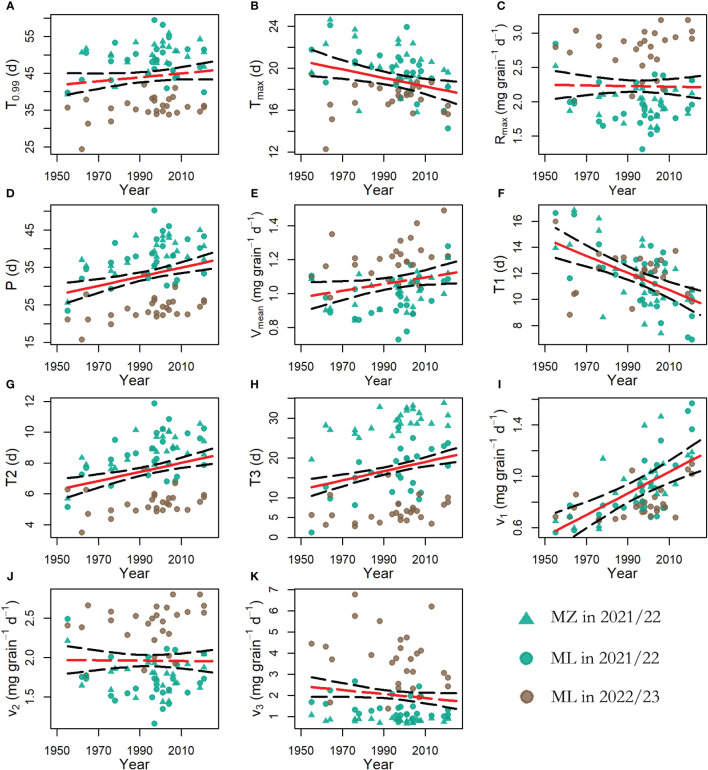
The relationships between winter wheat grain-filling parameters and year of release. The red solid lines represent significant relationships, whereas the dotted red lines represent non-significant relationships (*p* < 0.05). The black dotted lines represent the 95% confidence interval. The green triangles represent the wheat grain filling data from the MZ Experimental Station during the 2021-2022 growing season, the green circles represent the wheat grain filling data from the ML Experimental Station during the 2021-2022 growing season, and the brown circles represent the wheat grain filling data from the ML Experimental Station during the 2022-2023 growing season. **(A)** The relationship between the effective duration of grain-filling (T_0.99_) and the year of release. **(B)** The relationship between the time to reach the maximum grain-filling rate (T_max_) and the year of release. **(C)** The relationship between the maximum grain-filling rate (R_max_) and the year of release. **(D)** The relationship between the active grain-filling period (p) and the year of release. **(E)** The relationship between the average grain-filling rate (V_mean_) and the year of release. **(F)** The relationship between the duration of the initial grain-filling rate (T_1_) and the year of release. **(G)** The relationship between the duration of the rapid grain-filling rate (T_2_) and the year of release. **(H)** The relationship between the duration of the average grain-filling rate in the late grain-filling stage (T_3_) and the year of release. **(I)** The relationship between the average grain-filling rate at the initial grain-filling stage (v_1_) and the year of release. **(J)** The relationship between the average grain-filling rate of the rapid grain-filling stage (v_2_) and the year of release. **(K)** The relationship between the average grain-filling rate at the late grain-filling stage (v_3_) and the year of release.

To further demonstrate the relationship between wheat-filling parameters and year of release, RMLM was performed (**Figure 6**). During the year of release, *p*, *T*_2_, *T*_3_, and *v*_1_ increased significantly, whereas *T_max_* and *T*_1_ decreased significantly (*p* < 0.05). However, there were no significant relationships between *T*_0.99_, *R_max_*, *v_mean_*, *v*_2_, and *v*_3_ and the year of release (*p* > 0.05) (**Supplementary Table S2**). The results showed that the replacement of wheat cultivars in the Haihe Plain had an impact on grain-filling characteristics. Specifically, it has extended the active grain-filling period, particularly during the rapid filling stage, with durations of 5.7, 6.6, 7.1, 7.6, 7.7, 7.8, 8.2, and 8.0 days for the 1950s–2020s, respectively, and the late grain-filling stage, with durations of 8.9, 13.3, 15.8, 16.9, 17.7, 18.1, 19.0, and 20.0 days for the 1950s–2020s, respectively. Additionally, it has enhanced the average grain-filling rate, with values of 1.1, 1.0, 1.0, 1.1, 1.0, 1.0, 1.1, and 1.3 mg grain^−1^ d^−1^ for the 1950s–2020s, respectively. However, it has also shortened the duration of the initial grain-filling stage, with durations of 15.5, 13.6, 13.1, 12.3, 11.6, 11.4, 10.5, and 9.5 days for the 1950s–2020s, respectively.

## 4 Discussion

### 4.1 Grain yield and its components during wheat cultivar replacement

Domestic and foreign studies have shown that grain yield gradually increases with the replacement of the wheat crop in general; however, conclusions regarding yield components have been inconsistent (Zhou et al., 2007b; Feng et al., 2018; Qin et al., 2018, 2019; Sakuma and Schnurbusch, 2019). For foreign wheat cultivars, Sakuma and Schnurbusch (2019) showed that wheat yield was strongly influenced by changes in kernel number per spike; however, the effects of spike number and kernel weight were not significant. However, other studies have shown that a steady increase in the 1000-kernel weight and HI leads to a sustained increase in wheat yield and higher yield potential (Lo Valvo et al., 2018). Haihe Plain is located in northern China. In Northern China, Feng et al. (2018) reported that a significant increase in wheat yield was caused by an increase in kernel number per spike and kernel weight, although there was little change in spike number per unit area. Zhou et al. (2007a) also showed that there was a gradual increase in kernel weight during the replacement of wheat cultivars. Qin et al. (2015) showed that the replacement trends of wheat cultivars differed under different cultivation environments. Therefore, although previous studies have agreed that the replacement of wheat cultivars resulted in increased grain yield, differences in the timescales of studies and cultivation environments prevented consistent conclusions on the contribution of yield components to yield increases, but the increase in wheat yield in the Haihe Plain is closely related to kernel weight. This study demonstrated that both yield and grain weight increased significantly with the replacement of wheat cultivars. However, there are inconsistent conclusions regarding the reasons for the increase in yield during the process of cultivar replacement (Zhou et al., 2007b; Feng et al., 2018). In our study, the increase in kernel number was not significant, whereas kernel weight increased significantly during the cultivar replacement process. In contrast, Philipp et al. (2018) found that kernel number was the main reason for the increase in wheat yield. This discrepancy may be due to the different statistical methods used for measuring kernel number: the study used kernel number per square meter (Gonzalez-Navarro et al., 2016), while we measured kernel number per spike. Additionally, wheat cultivars have strong regional characteristics, which may also contribute to different reasons for yield increases during cultivar replacement in different areas. Given the significant increase in kernel weight observed in this study, it is necessary to further investigate the factors contributing to the increase in kernel weight during cultivar replacement.

Phenotype characteristics of wheat are influenced by both genetics and the environment; therefore, while wheat yield increased significantly with the year of release, there was also some influence of environmental factors, such as temperature, precipitation, and solar radiation, and soil factors such as topsoil thickness, soil water content, organic matter, nutrients, and trace elements in different ecological regions, differentially affecting the yield of the same wheat cultivar planted (Motzo and Giunta, 2004; Lobell et al., 2009; Subira et al., 2014). In the Haihe Plain, ML was located in the Heilonggang Plain zone, and MZ was located in the Piedmont plain of Mt. Taihang zone; the ecological conditions of the Piedmont plain for wheat planting were better than those of the Heilonggang Plain (Wu et al., 2023), which was further verified by comparison of soil characteristics in this study (**Supplementary Table S5**). Combining the two wheat growing seasons, the variance decomposition results showed that the spike number was more affected by the experimental year, which was due to the environmental factors of frost damage in the second year, which reduced the spike number compared to the first year, whereas the kernel weight was more affected by the year of release.

### 4.2 Grain-filling characteristics as year of release

For the wheat crop in general, Madani et al. (2010) showed that with the year of release, the increase in kernel weight was due to an increase in sink activity in modern wheat cultivars. Álvaro et al. (2008) found that the increase in the size of the sink in modern cultivars led to an increase in the capacity of the wheat stem to transport substances to the grain, thereby limiting the source capacity and lowering the kernel weight of early cultivars. Near the Haihe Plain, Zhou et al. (2007a) reported a significant increase in winter wheat yield caused by an increase in kernel weight. The mechanism was then established by Tian et al. (2011) who showed that the coordination of the source–sink relationship and increased pre- and post-anthesis transport capacity were the main reasons for the steady increase in kernel weight. Therefore, the kernel weight of modern wheat cultivars has increased because of the coordination of the source–sink relationship and the enhancement of sink activity in the Haihe Plain. In this study, the grain-filling rate gradually accelerated with the replacement of wheat cultivars in the Haihe Plain, which was consistent with previous studies. However, the grain-filling rate of wheat in the 1950s differed from that of other cultivars. It had a fast grain-filling rate but a short grain-filling duration, which may be attributed to the limitation of source capacity (Smith et al., 2018). Therefore, this study focused solely on sink activity and did not consider source capacity, which should be addressed in future research.

Furthermore, Khodarahmi et al. (2023) found that the average grain-filling rate increased gradually with the year of release; however, there was no significant association between grain-filling duration and the year of release. Other studies have also shown that the extension of grain-filling days is the main reason for higher kernel weights and yields of modern cultivars (Wiegand and Cuellar, 1981). For example, Wang and Shangguan (2015) showed that the key to increasing kernel weight is extending the duration of rapid and late grain-filling. The grain-filling rate in the rapid period was three times faster than that in the initial and late periods and, thus, had the greatest contribution to kernel weight (Xie et al., 2015). Wang et al. (2021) also showed that an increase in the grain-filling rate in the late period and a reduction in its fluctuation were key to increasing kernel weight, and the wheat grain-filling process is dominated by the regional ecological environment, especially temperature and light. Grain-filling characteristics vary depending on environmental conditions (Mirosavljević et al., 2018).

In the present study, with year of release, the duration of the initial grain-filling period became shorter whereas the filling rate increased, and the time to reach the maximum grain-filling rate advanced. Therefore, although the duration of the initial grain-filling stage of modern cultivars was shortened, the increase in filling rate resulted in an increase in kernel weight during the initial grain-filling stage. In addition, the significantly longer durations of the rapid and late grain-filling stages resulted in a longer active grain-filling period, which increased the kernel weight. This may be related to the fact that the North China Plain is often subjected to hot and dry winds, and the early cultivars are prone to early senescence in the middle and late filling stages (Cai et al., 2022). Furthermore, the Heilonggang Plain exhibited more rapid rates of grain-filling, including maximum and average rates, as well as during the rapid and late stages of grain-filling. In contrast, the Piedmont plain of Mount Taihang boasted longer durations for grain-filling periods, such as the effective grain-filling duration, the active grain-filling period, and the durations of both the rapid and late grain-filling stages. In addition, because of the lowest temperature of −9.3°C in the second year, which was close to the temperature of mild freezing damage (CMA, 2018), wheat suffered freezing damage, resulting in a shorter duration of filling period and active filling period in the second year. However, compared with the first year, the average and maximum filling rates increased. Beche et al. (2018) investigated the duration of developmental phases and eco-physiological traits related to grain yield in Brazilian wheat cultivars released across different decades, findings that suggest that longer durations in the growth and development stages can improve grain-filling characteristics and yield.

Kernel weight is one of the determinants of grain yield (Olmedo Pico et al., 2023). Maeoka et al. (2019) showed that the kernel weight of modern wheat cultivars increased by 27.28% and the spike number decreased by 14.83% compared with those of older cultivars. Similarly, Zhou et al. (2007a) showed that the kernel weight of modern cultivars in China increased by 49.31% and spike number decreased by 27.96% compared to those of earlier cultivars. Zheng et al. (2011) also showed that in the northern wheat region, the genetic gains for spike number, kernel number, and kernel weight were –0.62%, 0.75%, and 0.96%, respectively. In this study, kernel weight of modern cultivars reached 53.9 mg for Malan1, which was 26.7% higher than that in 1950s cultivars, but the spike number decreased gradually with the year of release, while modern cultivars decreased more rapidly by 62.6% compared to the 1950s. Sadras and Slafer (2012) showed that the number of spikes per unit area and kernel number exhibited greater phenotypic plasticity. Therefore, in the future, more attention should be paid to spike traits and canopy architecture.

## 5 Conclusion

The replacement of wheat cultivars in the Haihe Plain resulted in a significant increase in grain yield, in which the spike number decreased significantly and kernel weight increased significantly; however, there was no significant effect on kernel number. Additionally, with the release year, the duration of the initial grain-filling stage decreased, the rate increased, and the time to reach the maximum grain-filling rate advanced, whereas the kernel weight in the initial grain-filling stage increased. Moreover, owing to the extension of the duration of the rapid and late grain-filling stages, the active filling period was also significantly extended, which ultimately led to a larger kernel weight in modern cultivars. However, because of the large plasticity of spike and kernel numbers, they are also more sensitive to the environment than kernel weight, and their roles need to be emphasized in the selection of wheat cultivars, improvement of cultivation practices, and prevention of environmental disasters in the future.

## Data availability statement

The original contributions presented in the study are included in the article/**Supplementary Material**. Further inquiries can be directed to the corresponding authors.

## Author contributions

XZ: Investigation, Project administration, Writing – original draft. XL: Methodology, Software, Writing – original draft. LW: Investigation, Writing – original draft. QZ: Investigation, Writing – original draft. YY: Investigation, Writing – original draft. RD: Investigation, Writing – original draft. YX: Visualization, Writing – original draft. YW: Conceptualization, Formal analysis, Funding acquisition, Visualization, Writing – review & editing. WZ: Conceptualization, Funding acquisition, Writing – review & editing.

## Supplementary material

Supplementary Figure 1 Daily distribution of precipitation and temperature during the winter wheat growing seasons in 2021–2023.

Supplementary Figure 2 Fitted (lines) grain dry matter accumulation **(A)** and simulated grain-filling rate **(B)** in winter wheat under different years of release.

Supplementary Table 1 The results of linear mixed effects model of grain-filling characteristics. τ00: Founder parent; τ00: Founder parent: Cultivars; τ11: Cultivars; σ2: residuals; τ00: Founder parent: Cultivars; τ11: Cultivars; ICC: intraclass correlation coefficient; N1: number of random effects; N2: number of founder parent.

Supplementary Table 2 Fitted parameters of the logistic growth function for grain weight during the filling period for different cultivars of winter wheat in ML and MZ and anthesis dates of different wheat cultivars.

Supplementary Table 3 Grain-filling parameters for different winter wheat cultivars in ML and MZ. *T*_0.99_ is the effective duration of the grain-filling, *T_max_* is the time to reach the maximum grain-filling rate, *R_max_* is the maximum grain-filling rate, *p* is the active grain-filling period, and *V_mean_* is the average grain-filling rate.

Supplementary Table 4 The RDA ordination summary and first two ordination axes to quantitatively identify the relationships between winter wheat grain-filling characteristics with planting location and year of release and between grain yield and kernel weight with grain-filling characteristics.

Supplementary Table 5 The soil characteristics of the experimental stations in the 0- to 20-cm soil layer.

## References

[B1] Acreche M. M. Slafer G. A. (2009). Grain weight, radiation interception and use efficiency as affected by sink-strength in Mediterranean wheats released from 1940 to 2005. Field Crops Res. 110, 98–105. doi: 10.1016/j.fcr.2008.07.006

[B2] Aisawi K. A. B. Reynolds M. P. Singh R. P. Foulkes M. J. (2015). The physiological basis of the genetic progress in yield potential of CIMMYT spring wheat cultivars from 1966 to 2009. Crop Sci. 55, 1749–1764. doi: 10.2135/cropsci2014.09.0601

[B3] Álvaro F. Royo C. García del Moral L. F. Villegas D. (2008). Grain filling and dry matter translocation responses to source–sink modifications in a historical series of durum wheat. Crop Sci. 48, 1523–1531. doi: 10.2135/cropsci2007.10.0545

[B4] Asadi Zarch M. A. Sivakumar B. Sharma A. (2015). Droughts in a warming climate: A global assessment of Standardized precipitation index (SPI) and Reconnaissance drought index (RDI). J. Hydrology. 526, 183–195. doi: 10.1016/j.jhydrol.2014.09.071

[B5] Aziz M. M. Palta J. A. Siddique K. H. M. Sadras V. O. (2016). Five decades of selection for yield reduced root length density and increased nitrogen uptake per unit root length in Australian wheat varieties. Plant Soil 413, 181–192. doi: 10.1007/s11104-016-3059-y

[B6] Bates D. Mächler M. Bolker B. Walker S. (2015). Fitting linear mixed-effects models usinglme4. J. Stat. Software 67 (1), 1–48. doi: 10.18637/jss.v067.i01

[B7] Beche E. Benin G. da Silva C. L. Munaro L. B. Marchese J. A. (2014). Genetic gain in yield and changes associated with physiological traits in Brazilian wheat during the 20th century. Eur. J. Agron. 61, 49–59. doi: 10.1016/j.eja.2014.08.005

[B8] Beche E. Da Silva C. L. Todeschini M. H. Milioli A. S. Benin G. Marchese J. A. (2018). Improvement in Brazilian wheat breeding: changes in developmental phases and ecophysiological traits. Euphytica 214, 56. doi: 10.1007/s10681-018-2134-2

[B9] Borrás L. Slafer G. A. Otegui M. (2004). Seed dry weight response to source–sink manipulations in wheat, maize and soybean: a quantitative reappraisal. Field Crops Res. 86, 131–146. doi: 10.1016/j.fcr.2003.08.002

[B10] Dongyu C. Muhammad R. S. Yudong Z. Haibin T. Fanyu M. Haijun Y. (2022). Optimizing center pivot irrigation to regulate field microclimate and wheat physiology under dry-hot wind conditions in the North China plain. Water-Sui 14, 708. doi: 10.3390/w14050708

[B11] Cao G. (2001). North China Wheat (Beijing: China Agriculture Perss).

[B12] Chen X. Li K. Jia Y. (2008). Wheat in Hebei (Beijing: China Agricultural Science and Technology Press).

[B13] China Meteorological Administration(CMA) (2018). Indices for freezing injury of winter during wintering period in Huang-Huai-Hai Plain (Beijing: China Meteorological Press).

[B14] Dreccer M. F. Grashoff C. Rabbinge R. (1997). Source-sink ratio in barley (Hordeum vulgare L) during grain filling: Effects on senescence and grain protein concentration. Field Crops Res. 49, 269–277. doi: 10.1016/S0378-4290(96)01002-7

[B15] Fang Y. Liang L. Liu S. Xu B. Siddique K. H. Palta J. A.. (2021). Wheat cultivars with small root length density in the topsoil increased post-anthesis water use and grain yield in the semi-arid region on the Loess Plateau. Eur. J. Agron. 124, 126–143. doi: 10.1016/j.eja.2021.126243

[B16] Fang Q. Zhang X. Chen S. Shao L. Sun H. (2017). Selecting traits to increase winter wheat yield under climate change in the North China Plain. Field Crops Res. 207, 30–41. doi: 10.1016/j.fcr.2017.03.005

[B17] Feng W. Zhong M. Lemoine J. Biancale R. Hsu H. Xia J. (2013). Evaluation of groundwater depletion in North China using the Gravity Recovery and Climate Experiment (GRACE) data and ground-based measurements. Water Resour. Res. 49, 2110–2118. doi: 10.1002/wrcr.20192

[B18] Feng F. Han Y. Wang S. Yin S. Peng Z. Zhou M.. (2018). The effect of grain position on genetic improvement of grain number and thousand grain weight in winter wheat in North China. Front. Plant Sci. 9. doi: 10.3389/fpls.2018.00129 PMC5808325 29467787

[B19] Gonzalez-Navarro O. E. Griffiths S. Molero G. Reynolds M. P. Slafer G. A. (2016). Variation in developmental patterns among elite wheat lines and relationships with yield, yield components and spike fertility. Field Crops Res. 196, 294–304. doi: 10.1016/j.fcr.2016.07.019 28148999 PMC5268350

[B20] Hidaka K. Miyoshi Y. Ishii S. Suzui N. Yin Y. Kurita K.. (2019). Dynamic analysis of photosynthate translocation into strawberry fruits using non-invasive 11C-labeling supported with conventional destructive measurements using 13C-labeling. Front. Plant Sci. 9. doi: 10.3389/fpls.2018.01946 PMC6338039 30687351

[B21] Ho L. C. (1996). The mechanism of assimilate partitioning and carbohydrate compartmentation in fruit in relation to the quality and yield of tomato. J. Exp. Bot. 47, 1239–1243. doi: 10.1093/jxb/47.Special_Issue.1239 21245255

[B22] Hou M. Hao J. (2010). Research on strategic agricultural division and layout of the Huang-huai-hai Plain. Chin. J. Eco-Agriculture 18, 595–599. doi: 10.3724/SP.J.1011.2010.00595

[B23] Khodarahmi M. Soughi H. Shahbazi K. Jafarby J. Khavarinejad M. S. (2023). Trends in important agronomic traits, grain yield and its components in bread wheat cultivars released in northern warm and humid climate of Iran 1968–2018. Cereal Res. Commun. 51, 1003–1014. doi: 10.1007/s42976-023-00353-x

[B24] Li R. Q. (2014). Enetic analysis of winter wheat varieties and their super higher yield characters in hebei province: [dissertation thesis]. [Baoding (Hebei): University of Hebei Agriculture].

[B25] Liu X. Wang Y. Zhang Y. Ren X. Chen X. (2022). Can rainwater harvesting replace conventional irrigation for winter wheat production in dry semi-humid areas in China? Agric. Water Manage. 272, 107852. doi: 10.1016/j.agwat.2022.107852

[B26] Lobell D. B. Cassman K. G. Field C. B. (2009). Crop yield gaps: their importance, magnitudes, and causes. Annu. Rev. Environ. Resour. 34, 179–204. doi: 10.1146/annurev.environ.041008.093740

[B27] Lo Valvo P. J. Miralles D. J. Serrago R. A. (2018). Genetic progress in Argentine bread wheat varieties released between 1918 and 2011: Changes in physiological and numerical yield components. Field Crops Res. 221, 314–321. doi: 10.1016/j.fcr.2017.08.014

[B28] Ludlow M. M. Muchow R. C. (1990). A critical evaluation of traits for improving crop yields in water-limited environments. Adv. Agron. 43, 107–153. doi: 10.1016/S0065-2113(08)60477-0

[B29] Madani A. Rad A. S. Pazoki A. Nourmohammadi G. Zarghami R. (2010). Wheat (Triticum aestivum L.) grain filling and dry matter partitioning responses to source:sink modifications under postanthesis water and nitrogen deficiency. Acta Scientiarum. Agron. 32, 145–151.doi: 10.4025/actasciagron.v32i1.6273

[B30] Maeoka R. E. Sadras V. O. Ciampitti I. A. Diaz D. R. Fritz A. K. Lollato R. P. (2019). Changes in the phenotype of winter wheat varieties released between 1920 and 2016 in response to in-furrow fertilizer: biomass allocation, yield, and grain protein concentration. Front. Plant Sci. 10, 1786. doi: 10.3389/fpls.2019.01786 32082347 PMC7002544

[B31] Mirosavljević M. Momčilović V. Čanak P. Trkulja D. Mikić S. Jocković B. (2018). Grain filling variation in winter wheat, barley and triticale in pannonian environments. Cereal Res. Commun. 46, 697–706. doi: 10.1556/0806.46.2018.036

[B32] Motzo R. Giunta S. F. (2004). Relationship between grain yield and quality of durum wheats from different eras of breeding. Euphytica 140, 147–154. doi: 10.1007/s10681-004-2034-5

[B33] National Bureau of Statistics of China (NBS) (2022). China Statistical Yearbook (Beijing: China Statistics Press).

[B34] Oksanen J. Simpson G. Blanchet F. Kindt R. Legendre P. Minchin P.. (2023). vegan: Community Ecology Package. R package version 2.6-4 2022. Comprehensive R Archive Network.

[B35] Olmedo Pico L. B. Savin R. Schussler J. R. Vyn T. J. (2023). Late-vegetative and reproductive-stage nitrogen determinants of kernel weight in maize. Eur. J. Agron. 148, 126872. doi: 10.1016/j.eja.2023.126872

[B36] OrtizMonasterio J. I. Sayre K. D. Rajaram S. McMahon M. (1997). Genetic progress in wheat yield and nitrogen use efficiency under four nitrogen rates. Crop Sci. 37, 898–904. doi: 10.2135/cropsci1997.0011183X003700030033x

[B37] PhilippID N. Heiko W. Utkarsh B. Winfriede W. Albert Schulthess W. Weber H. (2018). Grain number and grain yield distribution along the spike remain stable despite breeding for high yield in winter wheat. PloS One 13 (10), e0205452. doi: 10.1371/journal.pone.0205452 30304020 PMC6179273

[B38] Qin X. Zhang F. Liu C. Yu H. Cao B. Tian S.. (2015). Wheat yield improvements in China: Past trends and future directions. Field Crops Res. 177, 117–124. doi: 10.1016/j.fcr.2015.03.013

[B39] Qin X. Li Y. Shi C. Song D. Wen X. Liao Y.. (2019). The number of cultivars in varietal winter-wheat mixtures influence aboveground biomass and grain yield in North China. Plant Soil 439, 131–143. doi: 10.1007/s11104-019-04084-z

[B40] Qin X. Feng F. Wen X. Siddique K. H. M. Liao Y. (2018). Historical genetic responses of yield and root traits in winter wheat in the yellow-Huai-Hai River valley region of China due to modern breeding, (1948–2012). Plant Soil 439, 7–18. doi: 10.1007/s11104-018-3832-1

[B41] Reynolds M. Bonnett D. Chapman S. C. Furbank R. T. Manès Y. Mather D. E.. (2011). Raising yield potential of wheat. I. Overview of a consortium approach and breeding strategies. J. Exp. Bot. 62, 439–452. doi: 10.1093/jxb/erq311 20952629

[B42] Sadras V. O. Slafer G. A. (2012). Environmental modulation of yield components in cereals: Heritabilities reveal a hierarchy of phenotypic plasticities. Field Crops Res. 127, 215–224. doi: 10.1016/j.fcr.2011.11.014

[B43] Sakuma S. Schnurbusch T. (2019). Of floral fortune: tinkering with the grain yield potential of cereal crops. New Phytol. 225, 1873–1882. doi: 10.1111/nph.16189 31509613

[B44] Shiferaw B. Smale M. Braun H. Duveiller E. Reynolds M. Muricho G. (2013). Crops that feed the world 10. Past successes and future challenges to the role played by wheat in global food security. Food Secur. 5, 291–317. doi: 10.1007/s12571-013-0263-y

[B45] Smith M. R. Rao I. M. Merchant A. (2018). Source-sink relationships in crop plants and their influence on yield development and nutritional quality. Front. Plant Sci. 9. doi: 10.3389/fpls.2018.01889 PMC6306447 30619435

[B46] Stoffel M. A. Nakagawa S. Schielzeth H. (2020). partR2: Partitioning R2 in generalized linear mixed models. bioRxiv 9, e11414. doi: 10.1101/2020.07.26.221168 PMC8162244 34113487

[B47] Subira J. Peña R. J. Álvaro F. Ammar K. Ramdani A. Royo C. (2014). Breeding progress in the pasta-making quality of durum wheat cultivars released in Italy and Spain during the 20th Century. Crop Pasture Sci. 65, 16–26. doi: 10.1071/CP13238

[B48] Thapa S. Xue Q. Jessup K. E. Rudd J. C. Liu S. Marek T. H. (2019). Yield determination in winter wheat under different water regimes. Field Crops Res. 233, 80–87. doi: 10.1016/j.fcr.2018.12.018

[B49] Tian Z. Jing Q. Dai T. Jiang D. Cao W. (2011). Effects of genetic improvements on grain yield and agronomic traits of winter wheat in the Yangtze River Basin of China. Field Crops Res. 124, 417–425. doi: 10.1016/j.fcr.2011.07.012

[B50] Wan W. Zhao Y. Li X. Xu J. Liu K. Guan S. (2022). A moderate reduction in irrigation and nitrogen improves water-nitrogen use efficiency, productivity, and profit under new type of drip irrigated spring wheat system. Front. Plant Sci. 13. doi: 10.3389/fpls.2022.1005945 PMC9589231 36299786

[B51] Wang L. Yu X. Gao J. Ma D. Li L. Hu S. (2021). Regulation of subsoiling tillage on the grain filling characteristics of maize varieties from different eras. Sci. Rep. 11, 20430. doi: 10.1038/s41598-021-99916-3 34650176 PMC8516906

[B52] Wang Y. Pan Y. Zhao F. Meng X. Li Q. Huang Y.. (2023). Changes in the lodging resistance of winter wheat from 1950s to the 2020s in Henan Province of China. BMC Plant Biol. 23 (1), 442. doi: 10.1186/s12870-023-04452-z 37726651 PMC10510142

[B53] Wang L. Shangguan Z. (2015). Photosynthetic rates and kernel-filling processes of big-spike wheat (Triticum aestivumL.) during the growth period. New Z. J. Crop Hortic. Sci. 43, 182–192. doi: 10.1080/01140671.2014.994644

[B54] Wickham H. (2016). ggplot2: Elegant Graphics for Data Analysis (New York: Springer-Verlag). doi: 10.1007/978-3-319-24277-4

[B55] Wiegand C. L. Cuellar J. A. (1981). Duration of grain filling and kernel weight of wheat as affected by temparature1. Crop Sci. 21, 95–101. doi: 10.2135/cropsci1981.0011183X001100010027x

[B56] Wu J. Gu Y. Wang N. Shen H. Ma X. (2023). Risk probability assessment of winter wheat net primary productivity loss and its driving factors in North China Plain. Field Crops Res. 300, 109013. doi: 10.1016/j.fcr.2023.109013

[B57] Xie P. Gao S. Sun F. (2019). An analysis of the decoupling relationship between CO2 emission in power industry and GDP in China based on LMDI method. J. Cleaner Prod. 211, 598–606. doi: 10.1016/j.jclepro.2018.11.212

[B58] Xie Q. Mayes S. Sparkes D. L. (2015). Carpel size, grain filling, and morphology determine individual grain weight in wheat. J. Exp. Bot. 66, 6715–6730. doi: 10.1093/jxb/erv378 26246614 PMC4623684

[B59] Zadoks J. C. Chang T. T. Konzak C. F. (1974). A decimal code for the growth stages of cereals. Weed Res. 14, 415–421. doi: 10.1111/j.1365-3180.1974.tb01084.x

[B60] Zhang H. Li T. Liu H. Mai C. Yu G. Li H.. (2020). Genetic progress in stem lodging resistance of the dominant wheat cultivars adapted to Yellow-Huai River Valleys Winter Wheat Zone in China since 1964. J. Integr. Agric. 19 (2), 438–448. doi: 10.1016/S2095-3119(19)62627-4

[B61] Zheng T. C. Zhang X. K. Yin G. H. Wang L. N. Han Y. L. Chen L.. (2011). Genetic gains in grain yield, net photosynthesis and stomatal conductance achieved in Henan Province of China between 1981 and 2008. Field Crops Res. 122, 225–233. doi: 10.1016/j.fcr.2011.03.015

[B62] Zheng X. Yu Z. Yu F. Shi Y. (2022). Grain-filling characteristics and yield formation of wheat in two different soil fertility fields in the Huang–Huai–Hai Plain. Front. Plant Sci. 13. doi: 10.3389/fpls.2022.932821 PMC9364837 35968109

[B63] Zhou Y. He Z. H. Sui X. X. Xia X. C. Zhang X. K. Zhang G. S.. (2007a). Genetic improvement of grain yield and associated traits in the northern China winter wheat region from 1960 to 2000. Crop Sci. 47, 245–253. doi: 10.2135/cropsci2006.03.0175

[B64] Zhou Y. Zhu H. Z. Cai S. B. He Z. H. Zhang X. K. Xia X. C.. (2007b). Genetic improvement of grain yield and associated traits in the southern China winter wheat region: 1949 to 2000. Euphytica 157, 465–473. doi: 10.1007/s10681-007-9376-8

[B65] Zhu Y. H. Weiner J. Yu M. X. Li F. M. (2018). Evolutionary agroecology: Trends in root architecture during wheat breeding. Evol. Appl. 12, 733–743. doi: 10.1111/eva.12749 30976306 PMC6439874

