# Supplementary figures and images for: Wheat yield and grain-filling characteristics due to cultivar replacement in the Haihe Plain in China
Source: Front Plant Sci. 2024 Jul 8;15:1374453. doi: 10.3389/fpls.2024.1374453 (PMC11260742; doi:10.3389/fpls.2024.1374453)

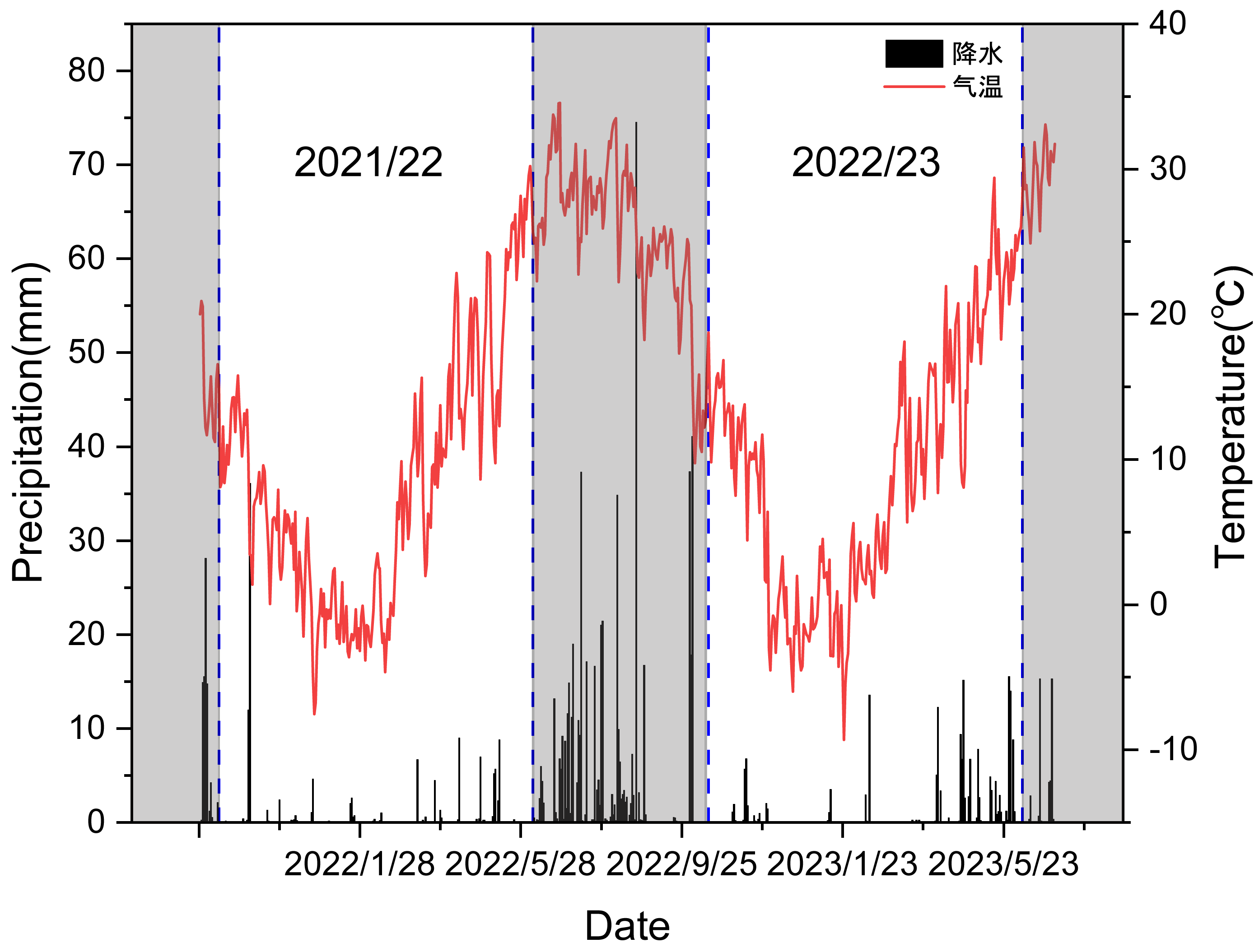

Supplement: Supplementary Figure 1 — Daily distribution of precipitation and temperature during the winter wheat growing seasons in 2021–2023. [file Image_1.tif]

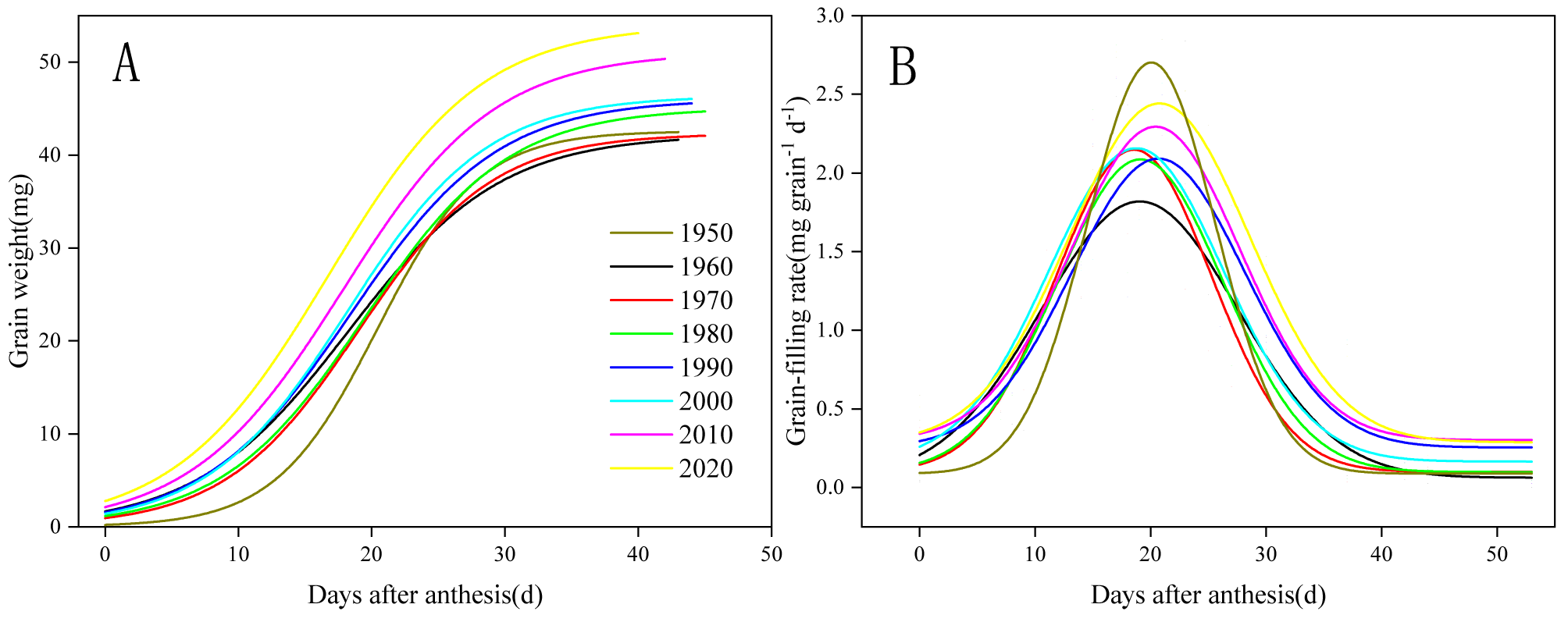

Supplement: Supplementary Figure 2 — Fitted (lines) grain dry matter accumulation (A) and simulated grain-filling rate (B) in winter wheat under different years of release. [file Image_2.tif]
