# Supplementary material for: Wheat yield and grain-filling characteristics due to cultivar replacement in the Haihe Plain in China
Source: Front Plant Sci. 2024 Jul 8;15:1374453. doi: 10.3389/fpls.2024.1374453 (PMC11260742; doi:10.3389/fpls.2024.1374453)
Supplement: Supplementary Table 1 — The results of linear mixed effects model of grain-filling characteristics. τ00: Founder parent; τ00: Founder parent: Cultivars; τ11: Cultivars; σ2: residuals; τ00: Founder parent: Cultivars; τ11: Cultivars; ICC: intraclass correlation coefficient; N1: number of random effects; N2: number of founder parent. [file Table_1.doc]

# Table S1. The results of linear mixed effects model of grain-filling characters.

| Parameters | Fix effects | | | | Random effects | | | | | | R2 | |
| --- | --- | --- | --- | --- | --- | --- | --- | --- | --- | --- | --- | --- |
|  | Intercept | Year | Site | Ex_year | σ2 | τ00 | τ11 | ICC | N1 | N2 | Marginal | Conditional |
| T_0.99_ | -69.52^ns^ | 0.05^ns^ | 1.39^ns^ | 12.47^***^ | 13.07 | 2.32 | 1.16 | 0.21 | 28 | 9 | 0.712 | 0.772 |
| T_max_ | 97.52^***^ | -0.04^***^ | 0.49^ns^ | 2.55^***^ | 3.29 | 0.00 | 0.29 |  | 29 | 9 | 0.410 |  |
| R_max_ | 3.67^ns^ | -0.00^ns^ | 0.05^ns^ | -0.80^***^ | 0.06 | 0.02 | 0.00 |  | 29 | 9 | 0.685 |  |
| p | -217.24^***^ | 0.12^***^ | 1.18^ns^ | 12.95^***^ | 14.09 | 3.10 | 0.00 |  | 28 | 9 | 0.769 |  |
| v_mean_ | -.2.67^ns^ | 0.00^ns^ | 0.03^ns^ | -0.23^***^ | 0.01 | 0.00 | 0.00 | 0.36 | 28 | 9 | 0.492 | 0.675 |
| T_1_ | 142.64^***^ | -0.07^***^ | 0.23^ns^ | -0.32^ns^ | 3.57 | 0.00 | 0.29 |  | 28 | 9 | 0.275 |  |
| v_1_ | -15.87^***^ | 0.01^***^ | 0.03^ns^ | 0.12^**^ | 0.02 | 0.00 | 0.01 | 0.34 | 28 | 9 | 0.461 | 0.644 |
| T_2_ | -52.79^***^ | 0.03^***^ | 0.26^ns^ | 3.07^***^ | 1.04 | 0.07 | 0.00 | 0.06 | 28 | 9 | 0.703 | 0.722 |
| v_2_ | 2.82^ns^ | -0.00^ns^ | 0.04^ns^ | -0.69^***^ | 0.05 | 0.02 | 0.00 |  | 28 | 9 | 0.680 |  |
| T_3_ | -224.38^***^ | 0.12^***^ | 12.47^***^ | 9.74^***^ | 13.41 | 0.54 | 0.00 |  | 28 | 9 | 0.869 |  |
| v_3_ | 22.68^*^ | -0.01^ns^ | -0.62^**^ | -2.25^***^ | 0.69 | 0.00 | 0.00 |  | 28 | 9 | 0.697 |  |

# Notes: τ00: Founder parent; τ00: Founder parent: Cultivars; τ11: Cultivars; σ2: residuals; τ00: Founder parent: Cultivars; τ11: Cultivars; ICC: Intraclass-Correlation Coefficient; N1: number of random effects; N2: number of founder parent.
