# Supplementary material for: Wheat yield and grain-filling characteristics due to cultivar replacement in the Haihe Plain in China
Source: Front Plant Sci. 2024 Jul 8;15:1374453. doi: 10.3389/fpls.2024.1374453 (PMC11260742; doi:10.3389/fpls.2024.1374453)
Supplement: Supplementary Table 2 — Fitted parameters of the logistic growth function for grain weight during the filling period for different cultivars of winter wheat in ML and MZ and anthesis dates of different wheat cultivars. [file Table_2.doc]

**Table S2. Fitted parameters of the Logistic growth function for grain weight during the filling period for different cultivars of winter wheat in ML and MZ and anthesis dates of different wheat cultivars.**

| Cultivars | Year | ML in 2021/22 | | | | | | MZ in 2021/22 | | | | | ML in 2022/23 | | | | | | | |
| --- | --- | --- | --- | --- | --- | --- | --- | --- | --- | --- | --- | --- | --- | --- | --- | --- | --- | --- | --- | --- |
|  |  | A | B | K | R^2^ | Anthesis date | A | | B | K | R^2^ | Anthesis  date | | | A | B | K | R^2^ | Anthesis date |  |
| Shijiazhuang407 | 1955 | 43.3 | 358.4 | 0.274 | 0.994 | 4.28 | 43.0 | | 97.8 | 0.234 | 0.999 | 5.3 | | | 39.5 | 247.8 | 0.283 | 0.979 | 4.27 |  |
| Shijiazhuang54 | 1964 | 42.2 | 60.7 | 0.176 | 0.974 | 4.28 | 47.6 | | 64.8 | 0.169 | 0.996 | 5.3 | | | 33.1 | 29.3 | 0.254 | 0.960 | 4.30 |  |
| Beijing8 | 1962 | 41.1 | 33.4 | 0.192 | 0.976 | 4.30 | 46.0 | | 37.2 | 0.162 | 0.999 | 5.2 | | | 28.6 | 106.8 | 0.380 | 0.993 | 4.28 |  |
| Jinan2 | 1965 |  |  |  |  |  |  | |  |  |  |  | | | 42.6 | 74.0 | 0.285 | 0.984 | 5.1 |  |
| Jimai1 | 1976 | 40.0 | 25.2 | 0.170 | 0.981 | 4.27 | 42.5 | | 49.7 | 0.170 | 0.990 | 5.3 | | | 38.9 | 157.0 | 0.303 | 0.987 | 4.28 |  |
| Jimai2 | 1976 | 44.0 | 70.2 | 0.208 | 0.989 | 4.26 | 46.9 | | 65.0 | 0.176 | 0.985 | 5.1 | | | 41.9 | 141.0 | 0.269 | 0.989 | 4.27 |  |
| Jimai3 | 1978 | 40.4 | 22.3 | 0.158 | 0.974 | 5.1 | 46.4 | | 17.8 | 0.181 | 0.977 | 5.6 | | |  |  |  |  |  |  |
| Taishan1 | 1984 | 40.6 | 27.0 | 0.173 | 0.977 | 4.30 | 50.6 | | 28.9 | 0.166 | 0.981 | 5.4 | | | 41.7 | 99.8 | 0.250 | 0.981 | 4.28 |  |
| Cang6001 | 1998 | 48.1 | 13.5 | 0.144 | 0.981 | 4.30 | 53.4 | | 12.2 | 0.147 | 0.985 | 5.6 | | | 46.1 | 91.4 | 0.262 | 0.994 | 4.28 |  |
| Jimai26 | 1988 | 44.2 | 30.4 | 0.166 | 0.988 | 4.26 | 48.7 | | 15.9 | 0.138 | 0.993 | 5.1 | | | 40.1 | 156.7 | 0.287 | 0.979 | 4.27 |  |
| Jimai36 | 1994 | 47.4 | 44.1 | 0.192 | 0.992 | 4.28 | 49.0 | | 33.9 | 0.179 | 0.984 | 5.3 | | | 42.7 | 190.8 | 0.296 | 0.954 | 4.28 |  |
| Han6172 | 2001 | 42.4 | 20.7 | 0.131 | 0.995 | 4.27 | 46.9 | | 14.1 | 0.138 | 0.98 | 5.2 | | | 43.6 | 70.6 | 0.244 | 0.987 | 4.28 |  |
| Heng4041 | 1997 | 45.2 | 24.2 | 0.172 | 0.983 | 4.28 | 51.3 | | 45.3 | 0.171 | 0.992 | 5.2 | | | 44.3 | 53.0 | 0.228 | 0.980 | 4.27 |  |
| Shi4185 | 1997 | 40.3 | 11.3 | 0.124 | 0.961 | 4.28 | 53.6 | | 32.2 | 0.153 | 0.972 | 5.3 | | | 41.0 | 54.3 | 0.223 | 0.988 | 4.27 |  |
| Jimai38 | 1996 | 41.1 | 16.8 | 0.16 | 0.985 | 5.1 | 52.8 | | 22.8 | 0.155 | 0.984 | 5.3 | | | 48.1 | 83.5 | 0.236 | 0.957 | 4.27 |  |
| Han4589 | 1998 | 43.9 | 28.8 | 0.166 | 0.994 | 4.28 | 48.9 | | 19.6 | 0.158 | 0.976 | 5.2 | | | 42.2 | 108.7 | 0.274 | 0.975 | 4.27 |  |
| Jimai30 | 1992 |  |  |  |  |  |  | |  |  |  |  | | 36.6 | | 23.5 | 0.230 | 0.955 | 5.1 |  |
| Heng95Guan26 | 2001 | 45.5 | 17.6 | 0.152 | 0.964 | 4.28 | 52.3 | | 20.6 | 0.142 | 0.993 | 5.3 | | 41.6 | | 115.5 | 0.269 | 0.983 | 4.27 |  |
| Shijiazhuang8 | 2003 | 46.9 | 34.7 | 0.202 | 0.989 | 5.1 | 51.4 | | 19.5 | 0.158 | 0.974 | 5.4 | | 48.4 | | 76.2 | 0.248 | 0.968 | 4.27 |  |
| Heng4399 | 2008 | 44.7 | 43.3 | 0.194 | 0.996 | 4.27 | 49.5 | | 19.1 | 0.157 | 0.970 | 5.2 | | 43.6 | | 92.6 | 0.266 | 0.994 | 4.27 |  |
| HengGuan35 | 2004 | 46.2 | 11.9 | 0.132 | 0.990 | 4.27 | 52.7 | | 13.3 | 0.127 | 0.982 | 5.2 | | 44.8 | | 71.2 | 0.234 | 0.983 | 4.26 |  |
| Jimai22 | 2006 | 47.5 | 13.5 | 0.164 | 0.990 | 5.1 | 51.3 | | 11.9 | 0.157 | 0.978 | 5.6 | | 49.5 | | 199.5 | 0.289 | 0.967 | 4.27 |  |
| Shiluan02-1 | 2004 | 40.4 | 35.7 | 0.178 | 0.983 | 4.27 | 46.6 | | 14.9 | 0.14 | 0.992 | 5.3 | | 36.3 | | 53.4 | 0.253 | 0.983 | 4.28 |  |
| Shimai15 | 2007 | 46.5 | 55.0 | 0.202 | 0.974 | 4.30 | 52.3 | | 16.0 | 0.144 | 0.962 | 5.6 | | 43.5 | | 176.4 | 0.272 | 0.958 | 4.28 |  |
| Shimai22 | 2013 | 46.4 | 13.5 | 0.144 | 0.982 | 5.1 | 54.7 | | 26.4 | 0.159 | 0.996 | 5.5 | | 41.5 | | 209.4 | 0.295 | 0.987 | 4.27 |  |
| Shinong086 | 2019 | 47.7 | 10.2 | 0.154 | 0.976 | 5.3 | 54.6 | | 14.1 | 0.133 | 0.974 | 5.3 | | 47.3 | | 72.0 | 0.287 | 0.964 | 4.30 |  |
| Malan1 | 2021 | 51.0 | 11.7 | 0.148 | 0.968 | 5.3 | 57.4 | | 18.0 | 0.148 | 0.982 | 5.5 | | 42.8 | | 78.8 | 0.319 | 0.985 | 5.1 |  |
| Malan6 | 2021 | 50.2 | 12.5 | 0.181 | 0.972 | 5.3 | 57.1 | | 19.4 | 0.162 | 0.972 | 5.6 | | 53.7 | | 69.0 | 0.243 | 0.969 | 4.28 |  |
