# Supplementary material for: Wheat yield and grain-filling characteristics due to cultivar replacement in the Haihe Plain in China
Source: Front Plant Sci. 2024 Jul 8;15:1374453. doi: 10.3389/fpls.2024.1374453 (PMC11260742; doi:10.3389/fpls.2024.1374453)
Supplement: Supplementary Table 3 — Grain-filling parameters for different winter wheat cultivars in ML and MZ. T 0.99 is the effective duration of the grain-filling, Tmax is the time to reach the maximum grain-filling rate, Rmax is the maximum grain-filling rate, p is the active grain-filling period, and Vmean is the average grain-filling rate. [file Table_3.doc]

**Table S3. Grain-filling parameters for different winter wheat cultivars in ML and MZ.**

| Cultivars | Year | ML in 2021/22 | | | | |  | MZ in 2021/22 | | | | |  | ML in 2022/23 | | | | |
| --- | --- | --- | --- | --- | --- | --- | --- | --- | --- | --- | --- | --- | --- | --- | --- | --- | --- | --- |
|  |  | T_0.99_ | T_max_ | R_max_ | p | V_mean_ |  | T_0.99_ | T_max_ | R_max_ | p | V_mean_ |  | T_0.99_ | T_max_ | R_max_ | p | V_mean_ |
| Shijiazhuang407 | 1955 | 39.73 | 21.78 | 2.84 | 23.43 | 1.11 |  | 39.18 | 19.57 | 2.52 | 25.61 | 1.09 |  | 35.67 | 19.45 | 2.80 | 21.17 | 1.10 |
| Shijiazhuang54 | 1964 | 50.55 | 24.10 | 1.97 | 34.53 | 0.89 |  | 51.73 | 24.62 | 2.01 | 35.41 | 0.91 |  | 37.82 | 16.52 | 2.01 | 27.80 | 0.98 |
| Beijing8 | 1962 | 43.22 | 18.66 | 1.99 | 32.07 | 0.98 |  | 50.65 | 22.31 | 1.87 | 37.00 | 0.90 |  | 31.28 | 15.13 | 3.03 | 21.09 | 1.35 |
| Jinan2 | 1965 |  |  |  |  |  |  |  |  |  |  |  |  | 24.37 | 12.29 | 2.72 | 15.78 | 1.16 |
| Jimai1 | 1976 | 48.24 | 20.44 | 1.71 | 36.30 | 0.85 |  | 50.12 | 23.03 | 1.80 | 35.37 | 0.84 |  | 31.89 | 16.71 | 2.94 | 19.82 | 1.21 |
| Jimai2 | 1976 | 43.13 | 20.67 | 2.29 | 29.33 | 1.03 |  | 49.75 | 23.68 | 2.07 | 34.04 | 0.93 |  | 35.50 | 18.41 | 2.82 | 22.32 | 1.17 |
| Jimai3 | 1978 | 53.38 | 21.56 | 1.65 | 41.55 | 0.85 |  | 41.25 | 15.89 | 2.10 | 33.12 | 1.11 |  |  |  |  |  |  |
| Taishan1 | 1984 | 48.68 | 20.11 | 1.75 | 37.31 | 0.89 |  | 47.93 | 20.26 | 2.10 | 36.14 | 1.05 |  | 36.84 | 18.44 | 2.60 | 24.03 | 1.12 |
| Cang6001 | 1998 | 51.54 | 18.96 | 1.80 | 42.54 | 0.98 |  | 48.43 | 17.07 | 1.96 | 40.94 | 1.09 |  | 34.76 | 17.23 | 3.02 | 22.89 | 1.31 |
| Jimai26 | 1988 | 50.31 | 21.22 | 1.82 | 37.97 | 0.91 |  | 53.41 | 20.08 | 1.68 | 43.52 | 0.90 |  | 35.15 | 18.11 | 2.88 | 22.25 | 1.20 |
| Jimai36 | 1994 | 44.72 | 20.10 | 2.28 | 32.15 | 1.08 |  | 45.31 | 19.66 | 2.19 | 33.49 | 1.07 |  | 41.95 | 16.56 | 2.08 | 33.16 | 1.08 |
| Han6172 | 2001 | 58.23 | 23.93 | 1.53 | 44.79 | 0.78 |  | 52.36 | 19.13 | 1.62 | 43.39 | 0.89 |  | 34.52 | 16.84 | 2.77 | 23.08 | 1.22 |
| Heng4041 | 1997 | 46.17 | 18.95 | 1.97 | 35.54 | 1.00 |  | 49.18 | 22.30 | 2.19 | 35.10 | 1.03 |  | 36.26 | 17.44 | 2.66 | 24.58 | 1.19 |
| Shi4185 | 1997 | 59.54 | 21.05 | 1.31 | 50.26 | 0.73 |  | 52.80 | 22.72 | 2.05 | 39.28 | 1.00 |  | 37.55 | 17.41 | 2.53 | 26.30 | 1.17 |
| Jimai38 | 1996 | 48.05 | 18.42 | 1.69 | 38.69 | 0.90 |  | 49.94 | 20.23 | 2.04 | 38.79 | 1.05 |  | 38.47 | 17.89 | 2.29 | 26.87 | 1.05 |
| Han4589 | 1998 | 48.71 | 20.74 | 1.88 | 36.53 | 0.93 |  | 47.77 | 18.77 | 1.94 | 37.86 | 1.01 |  | 38.14 | 18.71 | 2.84 | 25.37 | 1.25 |
| Jimai30 | 1992 |  |  |  |  |  |  |  |  |  |  |  |  | 33.93 | 17.13 | 2.89 | 21.93 | 1.23 |
| Heng95Guan26 | 2001 | 50.69 | 19.57 | 1.76 | 40.64 | 0.93 |  | 53.64 | 21.30 | 1.86 | 42.22 | 0.97 |  | 34.68 | 17.62 | 2.80 | 22.27 | 1.19 |
| Shijiazhuang8 | 2003 | 40.93 | 17.83 | 2.40 | 30.16 | 1.17 |  | 47.79 | 18.75 | 2.03 | 37.91 | 1.07 |  | 36.06 | 17.50 | 3.00 | 24.23 | 1.33 |
| Heng4399 | 2008 | 43.98 | 19.74 | 2.16 | 31.66 | 1.03 |  | 50.91 | 20.81 | 1.90 | 39.30 | 0.97 |  | 34.32 | 17.03 | 2.90 | 22.57 | 1.26 |
| HengGuan35 | 2004 | 54.86 | 19.59 | 1.59 | 46.04 | 0.88 |  | 55.91 | 22.36 | 1.78 | 43.81 | 0.92 |  | 37.92 | 18.25 | 2.62 | 25.68 | 1.17 |
| Jimai22 | 2006 | 45.80 | 16.58 | 1.95 | 38.15 | 1.07 |  | 45.02 | 15.78 | 2.02 | 38.18 | 1.13 |  | 38.51 | 18.70 | 3.19 | 25.87 | 1.42 |
| Shiluan02-1 | 2004 | 47.59 | 20.69 | 1.81 | 35.12 | 0.88 |  | 52.17 | 19.31 | 1.63 | 42.92 | 0.88 |  | 33.84 | 15.70 | 2.30 | 23.69 | 1.06 |
| Shimai15 | 2007 | 43.71 | 20.32 | 2.38 | 30.54 | 1.10 |  | 51.01 | 19.20 | 1.89 | 41.53 | 1.01 |  | 41.02 | 18.22 | 2.19 | 29.76 | 1.05 |
| Shimai22 | 2013 | 51.58 | 19.03 | 1.76 | 42.50 | 0.96 |  | 49.50 | 20.59 | 2.17 | 37.74 | 1.09 |  | 35.90 | 18.67 | 2.94 | 22.50 | 1.22 |
| Shinong086 | 2019 | 46.74 | 15.92 | 1.89 | 40.24 | 1.07 |  | 54.24 | 19.81 | 1.82 | 44.95 | 1.00 |  | 34.57 | 15.81 | 3.19 | 24.50 | 1.49 |
| Malan1 | 2021 | 51.45 | 18.25 | 1.95 | 43.36 | 1.09 |  | 50.73 | 19.60 | 2.12 | 40.66 | 1.12 |  | 35.74 | 15.62 | 2.92 | 26.27 | 1.42 |
| Malan6 | 2021 | 39.82 | 14.26 | 2.31 | 33.37 | 1.28 |  | 46.74 | 18.32 | 2.31 | 37.11 | 1.21 |  | 36.19 | 16.44 | 3.02 | 25.80 | 1.42 |

**Notes: *T_0.99_* is the effective duration of the grain-filling, *T_max_* is the time to reach the maximum grain-filling rate, *R_max_* is the maximum grain-filling rate, *p* is the active grain-filling period, *V_mean_* is the average grain-filling rate.**
