# Supplementary material for: Wheat yield and grain-filling characteristics due to cultivar replacement in the Haihe Plain in China
Source: Front Plant Sci. 2024 Jul 8;15:1374453. doi: 10.3389/fpls.2024.1374453 (PMC11260742; doi:10.3389/fpls.2024.1374453)
Supplement: Supplementary Table 4 — The RDA ordination summary and first two ordination axes to quantitatively identify the relationships between winter wheat grain-filling characteristics with planting location and year of release and between grain yield and kernel weight with grain-filling characteristics. [file Table_4.doc]

**Table S4. The RDA ordination summary and first two ordination axes to quantitatively identify the relationships between winter wheat grain-filling characters with planting location and year of release and between grain yield and kernel weight with grain-filling characters.**

| Wheat grain-filling characters with planting location and year of release | | | | Wheat grain yield and kernel weight with grain-filling characters | | | |
| --- | --- | --- | --- | --- | --- | --- | --- |
| Partitioning of correlations |  | Inertia | Proportion | Partitioning of correlations |  | inertia | Proportion |
|  | Total | 11.000 | 1.000 |  | Total | 2.000 | 1.000 |
|  | Constrained | 6.707 | 0.610 |  | Constrained | 1.447 | 0.723 |
|  | Unconstrained | 4.293 | 0.390 |  | Unconstrained | 0.553 | 0.277 |
| Eigenvalues, and their contribution to the correlations | | | | Eigenvalues, and their contribution to the correlations | | | |
|  | | RDA1 | RDA2 |  | | RDA1 | RDA2 |
| Eigenvalue | | 5.654 | 0.801 | Eigenvalue | | 1.350 | 0.096 |
| Proportion Explained | | 0.514 | 0.073 | Proportion Explained | | 0.675 | 0.048 |
| Cumulative Proportion | | 0.514 | 0.587 | Cumulative Proportion | | 0.675 | 0.723 |
| Dependent variables | T_0.99_ | -1.391 | -0.028 | Dependent variables | Grain yield | -1.774 | -0.614 |
|  | T_max_ | -0.873 | 0.466 |  | Kernel weight | -2.299 | 0.474 |
|  | R_max_ | 1.290 | -0.242 | Constraining variables | T_0.99_ | -0.535 | -0.339 |
|  | p | -1.391 | -0.215 |  | T_max_ | -0.162 | -0.032 |
|  | V_mean_ | 1.019 | -0.593 |  | R_max_ | 0.249 | 0.608 |
|  | T_1_ | 0.192 | 0.719 |  | p | -0.598 | -0.406 |
|  | v_1_ | -0.598 | -0.879 |  | V_mean_ | -0.073 | 0.551 |
|  | T_2_ | -1.364 | -0.212 |  | T_1_ | 0.320 | 0.314 |
|  | v_2_ | 1.286 | -0.256 |  | v_1_ | -0.683 | -0.095 |
|  | T_3_ | -1.369 | -0.208 |  | T_2_ | -0.584 | -0.375 |
|  | v_3_ | 1.358 | 0.055 |  | v_2_ | 0.240 | 0.605 |
| Constraining variables | Year | -0.140 | -0.976 |  | T_3_ | -0.676 | -0.491 |
|  | ML | 0.254 | 0.017 |  | v_3_ | 0.377 | 0.422 |
|  | MZ | -0.508 | -0.034 |  |  |  |  |
|  | 2021/22 | -0.440 | 0.051 |  |  |  |  |
|  | 2022/23 | 0.831 | -0.097 |  |  |  |  |
