# Supplementary material for: Wheat yield and grain-filling characteristics due to cultivar replacement in the Haihe Plain in China
Source: Front Plant Sci. 2024 Jul 8;15:1374453. doi: 10.3389/fpls.2024.1374453 (PMC11260742; doi:10.3389/fpls.2024.1374453)
Supplement: Supplementary Table 5 — The soil characteristics of the experimental stations in the 0- to 20-cm soil layer. [file Table_5.doc]

**Table S5. The soil characteristics of the experimental stations in 0–20 cm soil layer.**

| Experimental sites | Organic  matter  (g/kg) | Total nitrogen  (g/kg) | Alkaline  nitrogen  (mg/kg) | Available phosphorus  (mg/kg) | Available potassium  (mg/kg) |
| --- | --- | --- | --- | --- | --- |
| MZ | 22.4 | 1.26 | 131.2 | 25.8 | 122.2 |
| ML | 19.7 | 1.09 | 125.0 | 15.3 | 101.3 |
